# *De novo* transcriptome profile of coccolithophorid alga *Emiliania huxleyi* CCMP371 at different calcium concentrations with proteome analysis

**DOI:** 10.1371/journal.pone.0221938

**Published:** 2019-08-29

**Authors:** Onyou Nam, Jong-Moon Park, Hookeun Lee, EonSeon Jin

**Affiliations:** 1 Department of Life Science, Hanyang University, Seoul, Republic of Korea; 2 Gachon Institute of Pharmaceutical Sciences, Gachon College of Pharmacy, Gachon University, Incheon, Republic of Korea; Columbia University, UNITED STATES

## Abstract

The haptophyte alga *Emiliania huxleyi* is the most abundant coccolithophore in the modern ocean and produces elaborate calcite crystals, called coccolith, in a separate intracellular compartment known as the coccolith vesicle. Despite the importance of biomineralization in coccolithophores, the molecular mechanism underlying it remains unclear. Understanding this precise machinery at the molecular level will provide the knowledge needed to enable further manipulation of biomineralization. In our previous study, altering the calcium concentration modified the calcifying ability of *E*. *huxleyi* CCMP371. Therefore in this study, we tested *E*. *huxleyi* cells acclimated to three different calcium concentrations (0, 0.1, and 10 mM). To understand the whole transcript profile at different calcium concentrations, RNA-sequencing was performed and used for *de novo* assembly and annotation. The differentially expressed genes (DEGs) among the three different calcium concentrations were analyzed. The functional classification by gene ontology (GO) revealed that ‘intrinsic component of membrane’ was the most enriched of the GO terms at the ambient calcium concentration (10 mM) compared with the limited calcium concentrations (0 and 0.1 mM). Moreover, the DEGs in those comparisons were enriched mainly in ‘secondary metabolites biosynthesis, transport and catabolism’ and ‘signal transduction mechanisms’ in the KOG clusters and ‘processing in endoplasmic reticulum’, and ‘ABC transporters’ in the KEGG pathways. Furthermore, metabolic pathways involved in protein synthesis were enriched among the differentially expressed proteins. The results of this study provide a molecular profile for understanding the expression of transcripts and proteins in *E*. *huxleyi* at different calcium concentrations, which will help to identify the detailed mechanism of its calcification.

## Introduction

Coccolithophores are major biogenic calcite producers among the unicellular marine phytoplankton. Elaborate calcium carbonate scales called coccoliths are produced by these organisms. Each calcite crystal is formed under the stringent control of an intracellular biological system in a separate compartment known as the coccolith vesicle (CV) [1–4]. Among coccolithophores, *Emiliania huxleyi*, the most abundant calcite producer in the ocean, has been studied intensely [5]. However, the molecular mechanisms of its rigorous biomineralization system remain unclear.

Since the first expressed sequence tag (EST) analysis [6] was performed, several studies on *E*. *huxleyi* transcripts have varied the phosphate concentration to modulate the calcifying ability. The EST profile comparing calcifying and non-calcifying conditions was first to be analyzed [7]. Additionally, a suppressive subtraction hybridization and cDNA microarray were conducted to analyze differentially expressed genes (DEGs) and identify biomineralization-related genes in *E*. *huxleyi* [8, 9]. However, the *E*. *huxleyi* strain employed in previous reports was a non-calcifying strain that calcifies in phosphate-limited conditions. Moreover, the cells can be stressed in the phosphate-limited condition. Therefore, to alleviate the stress caused by phosphate-limitation, Quinn et al. (2006) compared calcifying and non-calcifying strains cultured in phosphate-replete conditions and analyzed DEGs, though those two strains are not isogenic [5]. Analyzing the haploid (non-calcifying) and diploid (calcifying) phases showed different gene expression profiles between the life cycle stages. More than 38,000 ESTs were sequenced to identify the genes associated with the specific processes of each life phase and understand the haploid phase of this organism [10]. To prevent ploidy-specific gene expression, Mackinder et al. (2011) compared the putative biomineralization gene expression of the calcifying and non-calcifying strains, which are both diploid strains and isogenic [11]. Previous studies have proposed many candidate genes related to the biomineralization of *E*. *huxleyi*. However, gene expression undergoes complex regulation by the molecular machinery of cells. Therefore, modifying the coccolith-forming ability of calcifying *E*. *huxleyi* will provide further information to understand the intricate mechanisms of coccolithogenesis.

Cultivating calcifying *E*. *huxleyi* cells at different calcium concentrations altered coccolithogenesis. Cells cultured under low calcium concentrations lacked the ability to form coccoliths compared with cells cultured in ambient calcium concentrations. However, the growth rate and photosynthetic efficiency of the cells were unaffected by their lack of calcifying ability [11–16]. In other words, calcium depletion in the culture medium produced a non-calcifying state, but cell growth was not affected by that event.

To investigate potential molecular mechanisms related to calcium in calcifying *E*. *huxleyi*, we used calcifying strain CCMP371, which calcifies constantly under phosphate-replete conditions. Nevertheless, this strain lost its coccolith-forming ability when calcium concentrations were limited. To understand the underlying molecular mechanism, we have analyzed the transcriptome of CCMP371 at different calcium concentrations. According to Read et al. (2013), the assembled reference *E*. *huxleyi* sequence analysis was performed using strain CCMP1516, which is known to calcify under phosphate-depleted conditions [7–9] and has recently lost its calcifying ability [17, 18]. Therefore, we have conducted a *de novo* assembly and analysis to analyze the molecular profile of *E*. *huxleyi* CCMP371 at different calcium concentrations. Additionally, based on *de novo* assembly, the transcriptome, combined with the proteome, was analyzed between calcifying and non-calcifying conditions. In this study, we elucidated the relevant pathways of *E*. *huxleyi* at different calcium concentrations at the transcript and protein levels.

## Materials and methods

### Algal strains and culture conditions

*E*. *huxleyi* strain CCMP371 was purchased from the Provasoli-Guillard National Center for Marine Algae and Microbiota. Cells were cultured in sterile artificial seawater [19] enriched with NaNO_3_, NaH_2_PO_4_∙H_2_O, trace metals, and vitamins at f/2 concentrations [20] with selenium (final conc. 0.01 μM) [21]. Cells were agitated twice a day by hand shaking and maintained at 20±1°C under irradiance of ~50 μmol m^-2^ s^-1^ (12:12 h light: dark). For the calcium-limited condition, cells cultivated at an ambient calcium concentration were centrifuged to remove the remaining medium before inoculating the cells in fresh medium without calcium ([Ca^2+^] 0 mM). Cells acclimated at [Ca^2+^] 0 mM were sub-cultured in fresh medium without calcium for more than 20 generations to completely remove the possibility of calcium in the medium.

### Cell growth and photosynthesis measurements

Cultures maintained in the exponential growth phase were used for inoculation. The number of cells was estimated by counting the cells using a hemocytometer (Marienfeld, Bad Mergentheim, Germany). For the experiments, cells were inoculated in 100 ml of fresh medium in 500 ml Erlenmeyer flasks at an initial cell concentration of 1 × 10^6^ cells ml^–1^. The maximum quantum yield of photosystem II (*F*_*v*_*/F*_*m*_) was measured at room temperature with a Walz image-PAM system M-series equipped with a CCD camera (Walz, Germany).

### Sample preparation and RNA sequencing

For all experiments, a 50 ml aliquot was collected in a conical tube from each replicate culture and concentrated by centrifugation (2,170x g, 10 min, 4°C; Hanil, Seoul, Korea) on day 4 of the growth phase. The pellet was immediately frozen by placing it into liquid nitrogen, and then it was stored at -80°C until further processing. The total RNA was extracted for transcriptomic analysis using a Hybrid-R^TM^ kit (GeneAll, Seoul, Korea) and DNase treatment, according to the manufacturer’s instructions. RNA quality and integrity were evaluated using an Agilent Technologies 2100 Bioanalyzer (Agilent Technologies, Santa Clara, CA, USA). Three samples per treatment (biological triplicate), for a total of nine samples (n = 9, 3 treatments x 3 replicates) were sent on dry ice to Macrogen (Seoul, Korea) for library construction and RNA sequencing (RNA-seq) using an Illumina HiSeq2500 (Illumina, Inc., San Diego, USA). The sequencing libraries were prepared according to the manufacturer’s instructions using the Illumina TruSeq SBS Kit v4 and sequenced in pair-end reads. Average of sequencing depth was 30 million reads (Table A in S1 File). Prior to *de novo* assembly, we used TopHat [22] to align the reads generated through RNA-seq to the *E*. *huxleyi* CCMP1516 draft genome sequence (v1.0) from the Joint Genome Institute. The raw read files were deposited in the NCBI Sequence Read Archive database under accession numbers SRR8501268, SRR8501269, SRR8501270, SRR8501271, SRR8501272, SRR8501273, SRR8501274, SRR8501275, and SRR8501276.

### Transcriptome assembly and annotation

The quality check on the raw sequences was done using FastQC (v0.11.7), and the trimming of reads was performed with Trimmomatic (v0.38) [23]. Trimmed reads from all of the samples were merged into one file to construct the transcriptome reference. The merged data were assembled using Trinity (r20140717) software [24] for *de novo* transcriptome assembly (kmer value of 25 with default parameters). All data were generated using the computing resources at Macrogen (Seoul, Korea). The assembled transcript fragments called contigs were further clustered into non-redundant transcripts using cd-hit-est (v4.6) [25] with a sequence identity threshold of 0.95. The assembled contigs were subjected to Benchmarking Using Single Copy Orthologues (BUSCO) (v.3.0.2) to assess transcriptome completeness using the eukaryota, (v20161102) dataset encompassing 303 genes [26]. Additionally, to estimate the map-back rate, we aligned the trimmed RNA-seq reads from each sample with the assembled reference using Bowtie (1.1.2) [27]. For comparison with the peptide sequenced data, TransDecoder (v3.0.1) (http://transdecoder.sf.net) was used to identify candidate coding regions within the generated transcriptome, looking for ORFs of at least 100 amino acids. For overall annotation of the unigenes, we searched the NCBI nucleotide (nt), non-redundant protein (nr), Pfam, and Uniprot databases using BLASTN from NCBI BLAST and BLASTX from DIAMOND (v0.9.21) [28] with an E-value cut-off of 1e^-5^. WebMGA [29] and BLAST (version 2.4.0, E-value cut-off of 1e^-5^) [30] were used for the EuKaryotic Orthologous Groups (KOG) and Kyoto Encyclopedia of Genes and Genomes (KEGG) annotations, respectively. In addition, Blast2GO [31] was used to generate GO annotations with nr annotations (E-value cut-off of 1e^-5^). This Transcriptome Shotgun Assembly project has been deposited at DDBJ/EMBL/GenBank under the accession GHJP00000000.

### Identification of differentially expressed genes and enrichment analysis

The RSEM algorithm (v1.2.29) was used to count the aligned reads and contigs with zero count in all 9 samples were removed from the analysis [32]. Thus, we conducted statistical analysis on 26,740 contigs, excluding 21,910 out of a total of 48,650 contigs. Data correction was performed using relative log expression normalization to reduce systematic bias, which could affect biological meanings when comparing samples using DESeq2 in the R environment [33]. The surrogate variable analysis method was used to correct for the batch effect (sva R library) [34]. Unigenes were sorted as DEGs if they showed a fold change of | log_2_FC | > 1 between [Ca^2+^] 0.1 vs 0 mM, [Ca^2+^] 10 vs 0 mM or [Ca^2+^] 10 vs 0.1 mM using a cut-off probability score to ensure an FDR (false positive rate) of less than 0.05. The GO enrichment analysis was performed using Blast2GO [31]. The KOG enrichment analysis was conducted by hypergeometric distribution testing using the Phyper function in the R software package (http://www.r-project.org/). Bonferroni correction was used to adjust the P-values. The significantly enriched functional clusters were selected based on a Q-value of less than 0.05. The KEGG pathway enrichment analysis was carried out using KOBAS 3.0 [35].

### Quantitative real-time polymerase chain reaction

Total RNA was isolated from cell cultures using a Hybrid-R kit (GeneAll, Korea). cDNA was synthesized using a Superscript III kit (Invitrogen, Carlsbad, CA, USA) primed with oligo dT primers, following the manufacturer’s protocol. The cDNA was used as a template for qPCR, which used SYBR green chemistry for amplicon detection. SYBR premix (Takara, Tokyo, Japan) and the Thermal Cycler Dice Real Time System TP8200 (Takara, Tokyo, Japan) were used for cDNA amplification. Actin was used as the reference gene in the qPCR [11]. Sequences of primers for the target and reference genes are provided in S1 File (Table B in S1 File).

### Protein digestion

*E*. *huxleyi* CCMP371 cells cultured in two different calcium concentrations (0.1 and 10 mM) were harvested by centrifugation. Biologically duplicated samples of each calcium concentration were used for analysis (n = 2). Protein was reduced with 5 mM TCEP (Thermo, USA) for 30 min at 37°C and alkylated by blocking cysteine residues with 15 mM IAA (Sigma Aldrich, St. Louis, MO, USA) at 25°C for 1 h in the dark. The pH was adjusted to 8 using 1M Tris (Sigma Aldrich, St. Louis, MO, USA), and the urea (Sigma Aldrich, St. Louis, MO, USA) concentration was reduced to less than 2 M using 10 mM Tris. The reduced and alkylated proteins were digested using sequence graded trypsin at a 1:50 ratio of protein: trypsin (Promega, Madison, WI, USA) at 37°C overnight. The activity of trypsin was stopped by adding formic acid (FA), and the pH was reduced to 2–3 before desalting. The digested peptides were desalted using C18 spin columns (Harvard Apparatus, Holliston, MA, USA), and the peptides were eluted with 80% acetonitrile in 0.1% formic acid (Honeywell, Charlotte, NC, USA) in water. All high-performance liquid chromatography–grade solvents were obtained from J.T Baker (Phillipsburg, NJ, USA).

### LC-MS/MS analysis

The prepared samples were resuspended in 0.1% formic acid in water and analyzed using a Q-Exactive Orbitrap hybrid mass spectrometer (Thermo Fisher Scientific, Waltham, MA, USA) along with an Ultimate 3000 system (Thermo Fisher Scientific, Waltham, MA, USA). We used a 2 cm x 75 μm ID trap column packed with 2 μm C18 resin and a 50 cm x 75 μm ID analytical column packed with 2 μm C18 resin to peptides depending on the peptides’ hydrophobicity. A data-dependent acquisition method was adopted, and the top 10 precursor peaks were selected and isolated for fragmentation. Ions were scanned in high resolution (70,000 in MS1, 17,500 in MS2 at m/z 400), and the MS scan range was 400–2,000 m/z at both the MS1 and MS2 levels. Precursor ions were fragmented with NCE (Normalized Collisional Energy) 27%. Dynamic exclusion was set to 30 s.

### Proteome data analysis

MS/MS data obtained from the Thermo Q-Exactive instrument were converted to mzXML using MSConvert for searching in Andromeda of MaxQuant (version 1.5.8.3). The mzXML files were searched using the following parameters- (a) enzyme: trypsin, (b) missed cleavage: 2, (c) fixed modification: carbamidomethyl (cysteine), (d) variable modification: oxidation (methionine), carbamyl (N-term), (e) precursor mass tolerance: up to 4.5ppm, (f) fragment mass tolerance: up to 20ppm. A cut-off probability score of less than 1% was used for FDR. Information about the identified peptides and proteins was aligned using the mass of charge state, retention time, and peak area. The information was normalized, and p-values and fold-change values were calculated using Perseus software (v1.5.8.5) for statistical analysis.

## Results

### *E*. *huxleyi* CCMP371 at different calcium concentrations

In our previous study [16], *E*. *huxleyi* CCMP371 cells cultured in 0 mM, 0.1 mM, and 10 mM (ambient) calcium concentrations showed no coccolith formation under limited-calcium conditions ([Ca^2+^] 0 and 0.1 mM). In the current study, however, the growth rate and *Fv/Fm* were not significantly affected by the different calcium concentrations (Table 1). We used calcifying ([Ca^2+^] 10 mM) and non-calcifying ([Ca^2+^] 0 and 0.1 mM) conditions in this study to unravel the underlying molecular profiles of biomineralization in *E*. *huxleyi* at different calcium concentrations.

**Table 1:** The growth rate and Fv/Fm measurements at different calcium concentrations.

	Ca^2+^ (mM)		
Factors	0	0.1	10
Growth rate (μ)	0.479±0.030	0.515±0.036	0.530±0.027
Fv/Fm	0.531±0.015	0.606±0.002	0.618±0.004

### *De novo* transcriptome sequencing, assembly, and annotation

In this study, we performed RNA-seq analysis of *E*. *huxleyi* CCMP371 cells cultured at different calcium concentrations (0, 0.1, and 10 mM). Three conditions with three biological replicates generated nine RNA-seq libraries. Because the mean mapping ratio of reads generated during this study to the *E*. *huxleyi* reference genome [*E*. *huxleyi* CCMP1516 (JGI)] was low (49.9%, Table C in S1 File), the libraries were combined and subjected to *de novo* transcriptome assembly. An average of 30,400,896 raw reads with approximately 52% GC contents were generated, which resulted in 30,159,074 reads after quality trimming (Table A in S1 File). The reads were assembled into 94,475 contigs, with an N50 contig length of 1,196 bp and an average length of 873.32 bp. For the assembled genes, the longest contigs (size cut-off > 201) were filtered and clustered to reduce the redundancy of the assembly. The number of contigs was thus decreased to 48,650, and those transcripts were defined as unigenes (Table 2, Table D in S1 File). Subsequently, the assembly was assessed by BUSCO analysis. Based on the 303 single copy orthologs for eukaryotes, 70.3% of the sequences were complete, with a small percentage of duplicated genes (2.3%). An additional 15.8% of orthologs were composed of fragmented genes, and the remaining 13.9% of the transcripts were missing from the transcriptome (S1A Fig). Furthermore, approximately 80% of our reads were aligned with the assembled reference (S1B Fig), suggesting the validity of the assembled transcriptome.

**Table 2:** Summary of RNA-sequencing and *de novo* assembly.

Assembly	Unigene
Total number of unigenes	48,650
GC content (%)	67.35
N50 (bp)	1,172
Maximum unigene size (bp)	17,967
Minimum unigene size (bp)	201
Median unigene length (bp)	594
Average unigene length (bp)	826.36

The overall annotated ratio for the assembly was 75.52%, with 36,471 of the 48,650 unigenes annotated in at least one public database (Table 3, Table E in S1 File). Of the total 48,650 unigenes, 23,831 unigenes were assigned to GO classes with functional terms (S2 Fig). For additional functional prediction and classification, unigenes were analyzed against the KOG database (S3 Fig). The 10,692 unique sequences were divided into 26 KOG categories. A total of 4,700 unigenes were further analyzed for functional classification using the KEGG pathways (S4 Fig).

**Table 3:** Annotation of unigenes against public databases.

Annotated	Number of	Annotated unigene
Database	annotated unigenes	ratio (%)
Nr	32,442	66.68
GO	23,831	48.98
Uniprot	13,281	27.30
Pfam	12,388	25.46
Nt	31,127	63.98
KOG	10,692	21.98
KEGG	20,281	41.69
All	36,471	75.52

### Differentially expressed genes at different calcium concentrations

**Fig 1:**
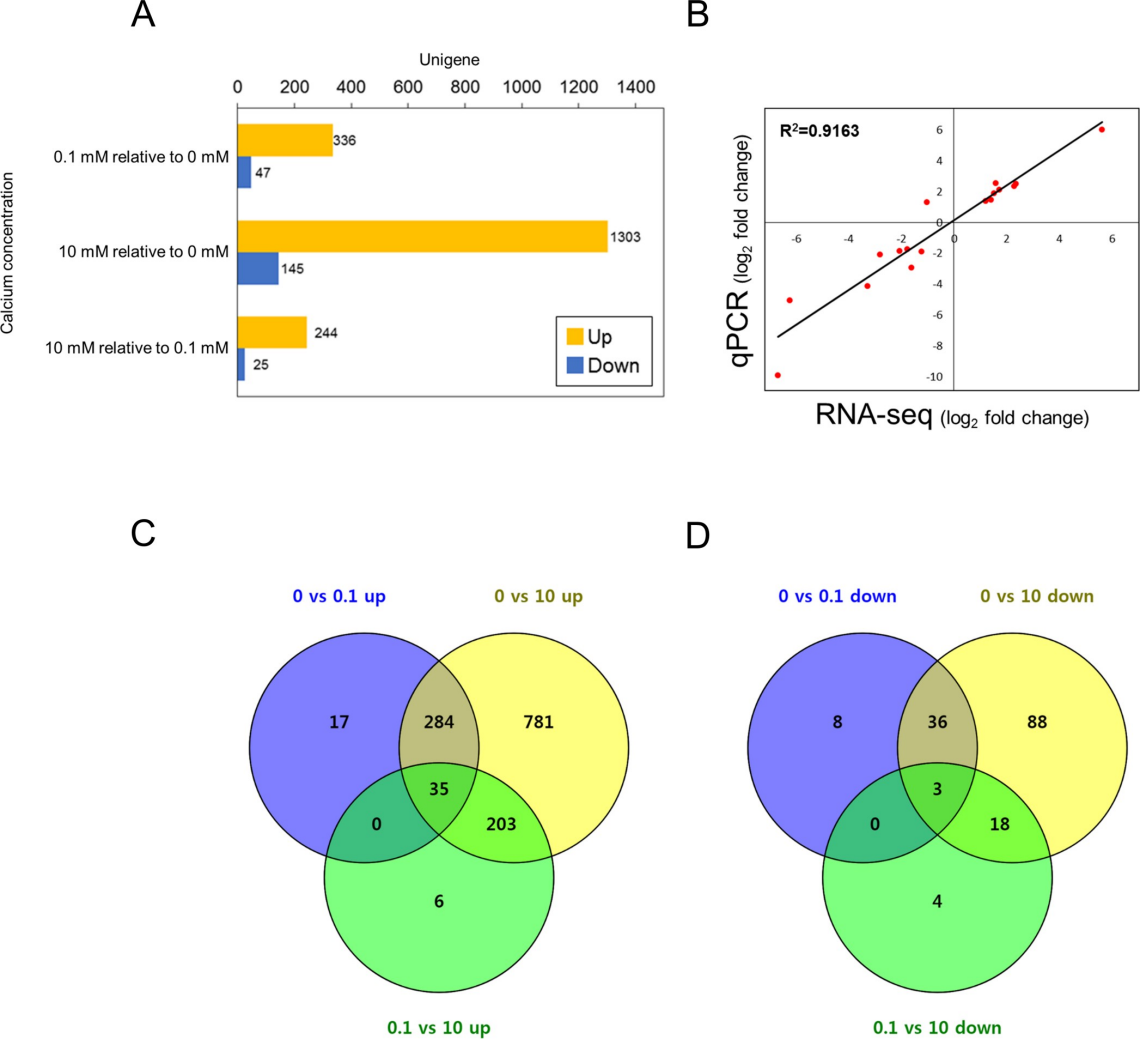
Differentially expressed genes (DEGs) involved in *E*. *huxleyi* at different calcium concentrations. (A) DEGs from each comparison of different calcium concentrations. Bar charts show up-regulated and down-regulated DEGs in yellow and blue, respectively. (B) Correlation between RNA-seq data and qPCR analysis for validation. (C, D) DEG comparison of the (C) up- and (D) down-regulated groups. The Venn diagram was drawn by Venny^2.1^ [36].

The DEGs, | log_2_ FC | > 1 (FDR<0.05), were analyzed comparing [Ca^2+^] 0.1 vs 0 mM, [Ca^2+^] 10 vs 0 mM, and [Ca^2+^] 10 vs 0.1 mM (S5 Fig, Table F in S1 File). In [Ca^2+^] 0.1 mM relative to [Ca^2+^] 0 mM, 336 genes were up-regulated, and 47 genes were down-regulated. In [Ca^2+^] 10 mM compared to [Ca^2+^] 0.1 mM, 244 up-regulated and 25 down-regulated genes were identified. The comparison between [Ca^2+^] 10 mM and [Ca^2+^] 0 mM showed the largest number of DEGs, with 1,303 up-regulated and 145 down-regulated in 10 mM calcium (Fig 1A). The three DEG comparisons were compared by Venn diagrams. In the up-regulated Venn diagram, 781 and 6 genes were up-regulated in 10 mM calcium compared to [Ca^2+^] 0 mM and [Ca^2+^] 0.1 mM, respectively. A total of 203 DEGs were commonly up-regulated in 10 mM calcium at both [Ca^2+^] 10 vs 0 mM and [Ca^2+^] 10 vs 0.1 mM (Fig 1C). Among the down-regulated DEGs, 88 and 4 genes were down-regulated in 10 mM calcium compared with [Ca^2+^] 0 mM and [Ca^2+^] 0.1 mM, respectively. A total of 18 DEGs were commonly down-regulated in 10 mM calcium compared with both [Ca^2+^] 0 mM and [Ca^2+^] 0.1 mM (Fig 1D). We randomly selected 17 up-regulated and down-regulated DEGs to validate our transcriptome assembly and analysis using real-time qPCR. Of the 17 unigenes, 16 showed expression patterns similar to those observed in the RNA-seq results (Fig 1B).

### Expression of biomineralization-related genes at different calcium concentrations

**Fig 2:**
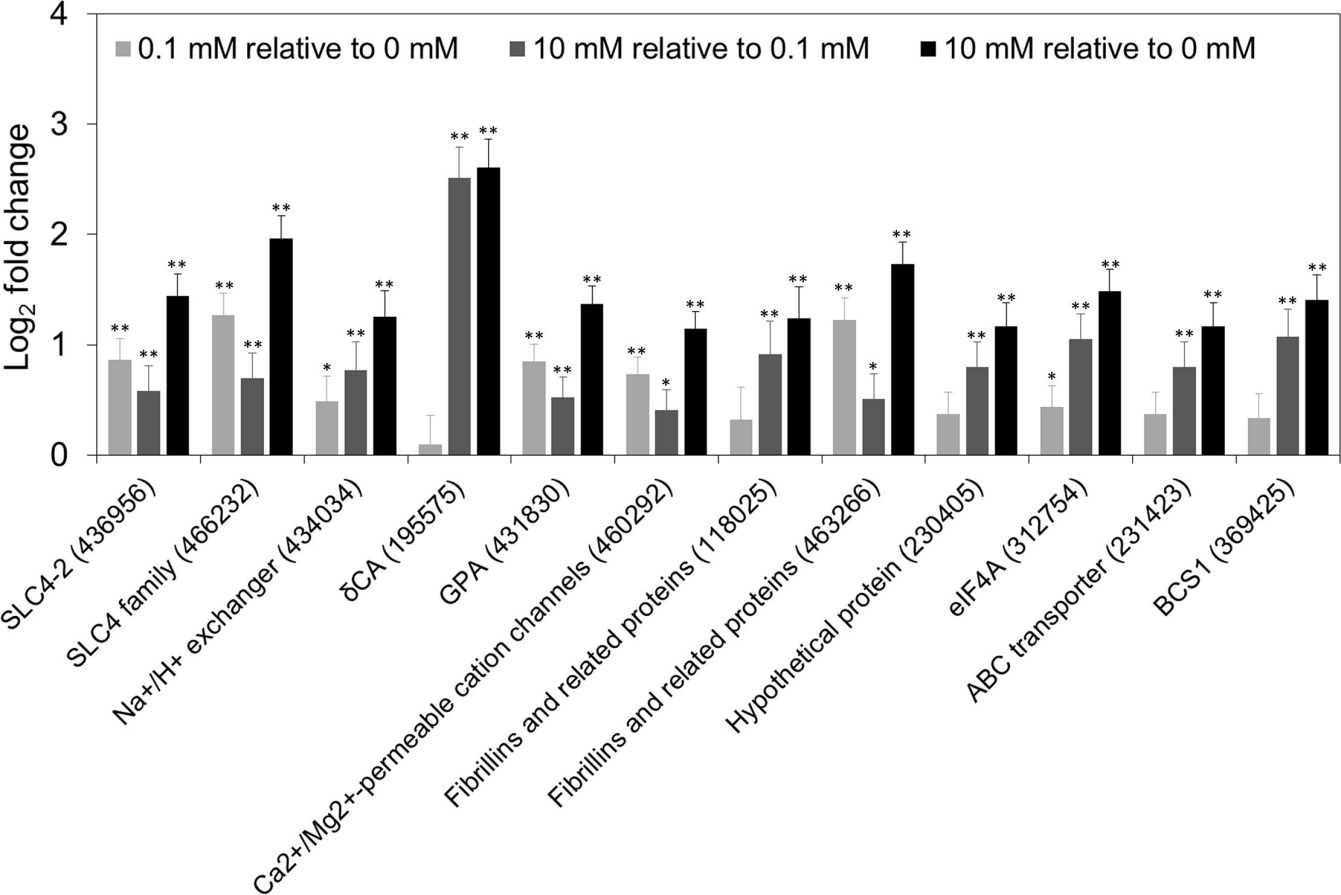
Putative biomineralization-related genes in DEGs at different calcium concentrations. The log_2_ fold changes of the genes are shown as a bar chart (log_2_FC; ± SE, light gray: [Ca^2+^] 0.1 mM relative to [Ca^2+^] 0 mM, dark gray: [Ca^2+^] 10 mM relative to [Ca^2+^] 0.1 mM, black: [Ca^2+^] 10 mM relative to [Ca^2+^] 0 mM). The gene IDs in the bar chart are in the following order: SLC4-2 (c31857_g11_i1), SLC family (c25647_g1_i1), Na^+^/H^+^ exchanger (c31114_g3_i2), delta CA (c24914_g1_i2), calcium-binding GPA (glutamic acid, proline and alanine) protein (c20552_g1_i1), Ca^2+^/Mg^2+^ permeable cation channels (LTRPC family) (c30953_g1_i11), fibrillins and related proteins containing a Ca^2+^-binding epidermal growth factor (EGF)-like domain (JGI ID 118025; c28155_g1_i1), fibrillins and related proteins containing Ca^2+^-binding EGF-like domain (JGI ID 463266; c28980_g1_i1), hypothetical protein (c34539_g1_i1), eukaryotic initiation factor 4A (eIF4A) (c22513_g1_i1), ABC transporter (c34539_g1_i1), and putative mitochondrial chaperone BCS1 (c23862_g2_i1). Asterisks represent significant expression change (*p < 0.05, **p < 0.005).

Within the DEGs, we examined genes previously reported as biomineralization-related genes in *E*. *huxleyi* [10, 11, 37–39]. Potential genes were separated, as Benner et al. (2013) categorized, into groups for calcium binding and transport, carbonic anhydrases (CAs), inorganic carbon transport, and pH homeostasis. Putative genes were analyzed by BLAST search for homologs from the transcriptome (Table G in S1 File). Among the previously reported biomineralization-related genes, 12 genes were differentially expressed in [Ca^2+^] 10 mM relative to [Ca^2+^] 0 mM (Fig 2). These DEGs, including Ca^2+^/Mg^2+^ permeable cation channels (LTRPC family) (JGI ID 460292), fibrillins and related proteins containing a Ca^2+^-binding epidermal growth factor (EGF)-like domain (JGI ID 118025, 463266), calcium-binding GPA (glutamic acid, proline, and alanine) protein (JGI ID 431830), delta CA (JGI ID 195575), anion exchanger-like, SLC4 Na^+^ independent Cl^-^/HCO_3_^-^exchangers (JGI ID 436956, 466232), and eukaryotic Na^+^/H^+^ exchanger (JGI ID 434034) were up-regulated in [Ca^2+^] 10 mM compared with [Ca^2+^] 0 mM. Additionally, we investigated the gene expression of putative biomineralization-associated genes from our previous study (Fig 2) [16]. The hypothetical protein (JGI ID 230405), eukaryotic initiation factor 4A (eIF4A) (JGI ID 312754), putative mitochondrial chaperone BCS1 (JGI ID 369425), and putative ABC transporter (JGI ID 231423) were up-regulated DEGs in [Ca^2+^] 10 mM relative to [Ca^2+^] 0 mM. Furthermore, eIF4A and putative mitochondrial chaperone BCS1 were up-regulated DEGs in [Ca^2+^] 10 mM compared with [Ca^2+^] 0.1 mM. However, the putative genes for Ca^2+^/H^+^ exchangers 3 and 4, ER-type Ca^2+^-ATPase2, anion exchanger like 1 (AEL1), gamma carbonic anhydrase (γCA), and vacuolar H^+^-ATPase (ATPVc/c’), which are known to be related to the biomineralization process, were not differentially expressed at the different calcium concentrations.

### Gene Ontology enrichment analysis

**Fig 3:**
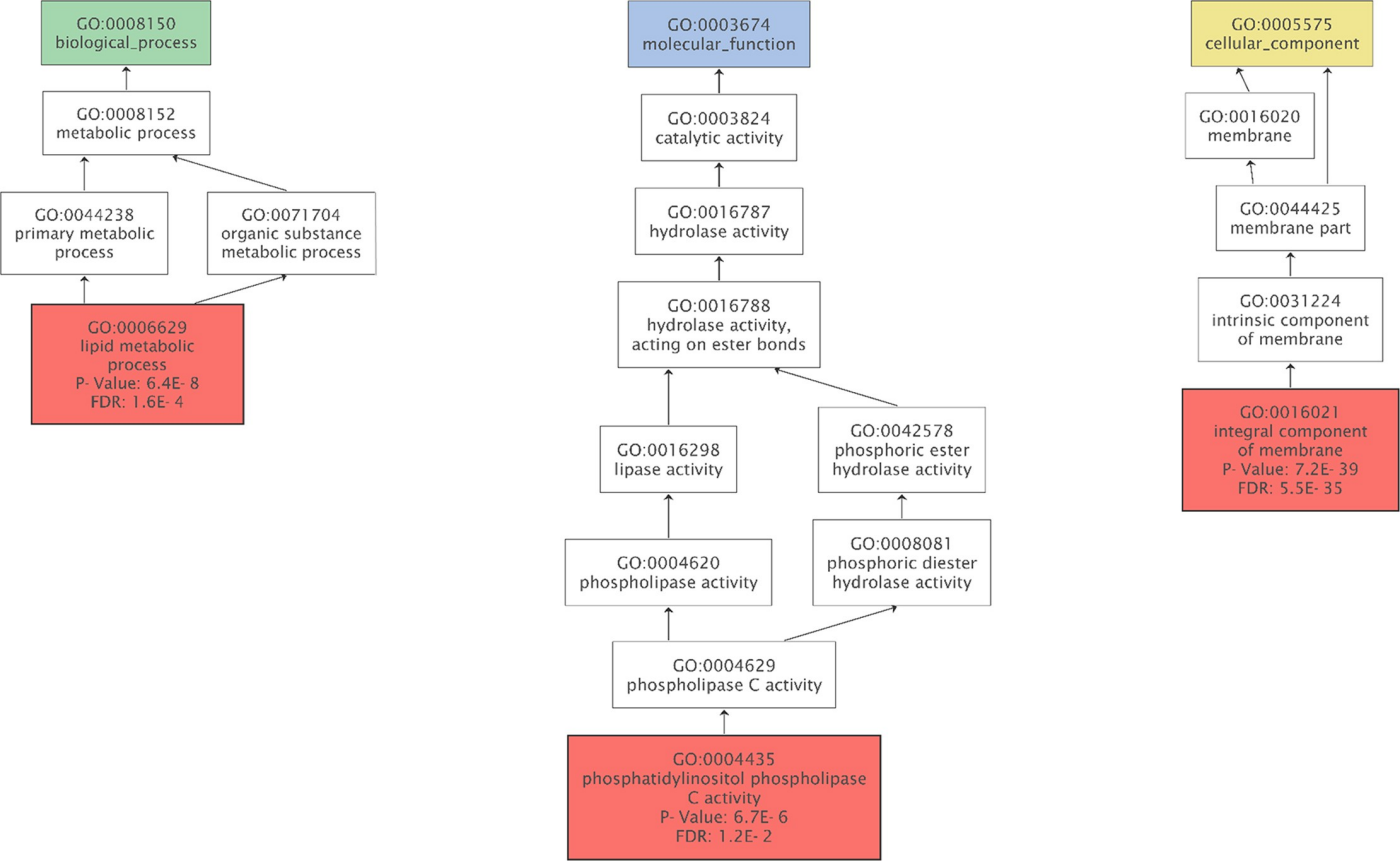
Gene Ontology (GO) enrichment analysis of DEGs up-regulated in [Ca^2+^] 10 mM relative to [Ca^2+^] 0 mM. The GO terms were enriched using Blast2GO. The enriched terms were reduced to most specific and shown in the red box (cut-off: FDR<0.05, green: biological process, blue: molecular function, yellow: cellular component).

To further examine the functional classification of the transcriptome, we performed a GO analysis. To compare the ambient-condition with the limited calcium conditions, DEG groups were analyzed (S6A and S6B Fig). In both DEG comparisons, the largest number of unigenes was grouped in the ‘integral component of membrane’ of cellular component category. The GO term enrichment was performed in up-regulated DEGs in [Ca^2+^] 10 mM relative to [Ca^2+^] 0 mM. The 1,303 unique, statistically significant DEGs were analyzed by Fisher’s exact test with an FDR threshold of 0.05 (Fig 3). Among the DEGs up-regulated in [Ca^2+^] 10 mM relative to [Ca^2+^] 0 mM, three sub-categories from three main GO categories were found to be enriched. The most frequent GO term was integral component of membrane followed by lipid metabolic process. For the down-regulated DEGs in [Ca^2+^] 10 mM relative to [Ca^2+^] 0 mM and [Ca^2+^] 0.1 mM, no GO terms were enriched with an FDR threshold of 0.05.

### KOG enrichment analysis

A total of 352 DEGs up-regulated in [Ca^2+^] 10 mM relative to [Ca^2+^] 0 mM were functionally classified into 23 KOG clusters. The genes associated with ‘secondary metabolites biosynthesis, transport and catabolism’ and ‘signal transduction mechanisms’ were over-represented in [Ca^2+^] 10 mM relative to [Ca^2+^] 0 mM (Table 4). However, no significantly enriched functional clusters, based on a q-value less than 0.05, were observed among the down-regulated DEGs in [Ca^2+^] 10 mM relative to [Ca^2+^] 0 mM.

**Table 4:** KOG enrichment analysis of DEGs compared with the transcriptome. The up-regulated DEGs in [Ca^2+^] 10 mM relative to [Ca^2+^] 0 mM were analyzed.

KOG functional cluster	DEG frequency of use	Transcriptome frequency of use	P-value	Q-value
Secondary metabolites biosynthesis, transport, and catabolism	23 of 353 (6.52%)	265 of 10692 (2.48%)	7.16E-06	1.86E-04
Signal transduction mechanisms	55 of 353 (15.58%)	1062 of 10692 (9.93%)	2.60E-04	0.007
Extracellular structures	4 of 353 (1.13%)	43 of 10692 (0.40%)	0.013	0.341
Lipid transport and metabolism	29 of 353 (8.22%)	613 of 10692 (5.73%)	0.020	0.512
Inorganic ion transport and metabolism	11 of 353 (3.12%)	242 of 10692 (2.26%)	0.105	1
Cell wall/membrane/envelope biogenesis	12 of 353 (3.40%)	278 of 10692 (2.60%)	0.131	1
Defense mechanisms	4 of 353 (1.13%)	86 of 10692 (0.80%)	0.155	1
Chromatin structure and dynamics	8 of 353 (2.27%)	214 of 10692 (2.00%)	0.276	1
Intracellular trafficking, secretion, and vesicular transport	14 of 353 (3.97%)	402 of 10692 (3.76%)	0.350	1
Translation, ribosomal structure and biogenesis	17 of 353 (4.82%)	503 of 10692 (4.70%)	0.396	1
General function prediction only	51 of 353 (14.45%)	1537 of 10692 (14.38%)	0.447	1
Cytoskeleton	8 of 353 (2.27%)	254 of 10692 (2.38%)	0.463	1
Carbohydrate transport and metabolism	14 of 353 (3.97%)	439 of 10692 (4.11%)	0.484	1
Function unknown	20 of 353 (5.67%)	655 of 10692 (6.13%)	0.589	1
Energy production and conversion	12 of 353 (3.40%)	415 of 10692 (3.88%)	0.618	1
Cell cycle control, cell division, chromosome partitioning	3 of 353 (0.85%)	131 of 10692 (1.23%)	0.633	1
Amino acid transport and metabolism	14 of 353 (3.97%)	488 of 10692 (4.56%)	0.651	1
Transcription	11 of 353 (3.12%)	435 of 10692 (4.07%)	0.779	1
Nucleotide transport and metabolism	2 of 353 (0.57%)	141 of 10692 (1.32%)	0.849	1
Coenzyme transport and metabolism	2 of 353 (0.57%)	156 of 10692 (1.46%)	0.893	1
Posttranslational modification, protein turnover, chaperones	35 of 353 (9.92%)	1362 of 10692 (12.74%)	1	1
Replication, recombination, and repair	2 of 353 (0.57%)	388 of 10692 (3.63%)	1	1
RNA processing and modification	2 of 353 (0.57%)	564 of 10692 (5.27%)	1	1

### KEGG pathway enrichment analysis

**Fig 4:**
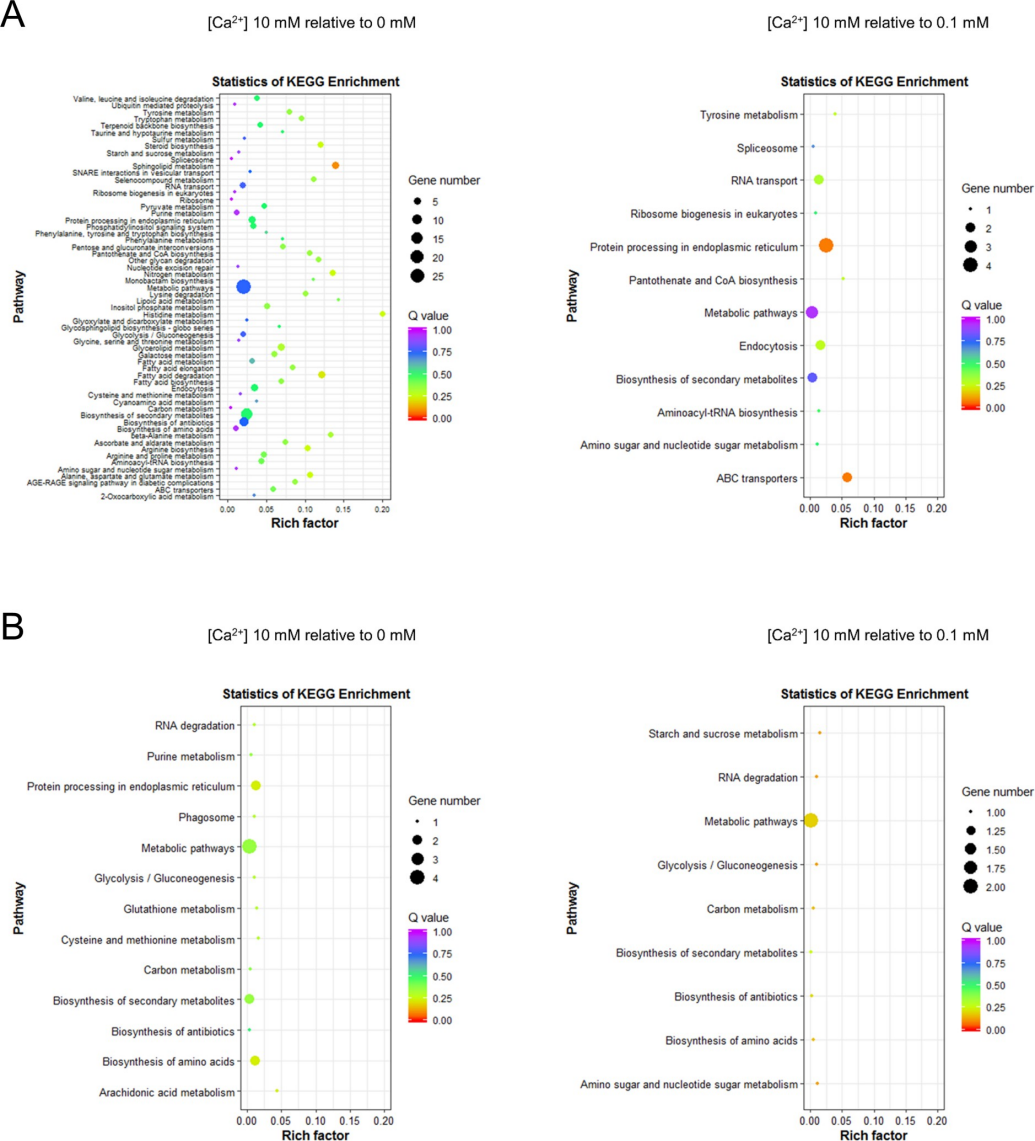
KEGG pathway enrichment of DEGs at different calcium concentrations. The pathways enriched in (A) up-regulated and (B) down-regulated DEGs in the comparison between [Ca^2+^] 10 vs 0 mM and [Ca^2+^] 10 vs 0.1 mM.

To further analyze the metabolic pathways involved at the different calcium concentrations, we completed a KEGG enrichment analysis on two DEG groups in the ambient- and limited-calcium conditions. Additionally, we increased the DEG criteria to examine the genes putatively related to the biomineralization process. The cut-off for the comparison between [Ca^2+^] 0.1 vs 0 mM was | log_2_ FC | < 1, and this criterion was added to the DEGs. As to investigate the genes that were not differentially expressed between the limited-calcium concentrations ([Ca^2+^] 0.1 vs 0 mM) and up- or down-regulated DEGs in [Ca^2+^] 10 mM relative to limited-calcium concentrations. The KEGG enrichment analysis was performed in two DEG groups ([Ca^2+^] 10 vs 0 mM and [Ca^2+^] 10 vs 0.1 mM). In the pathways up-regulated in [Ca^2+^] 10 mM relative to [Ca^2+^] 0mM, various pathways were enriched, including sphingolipid metabolism, histidine metabolism, and nitrogen metabolism (Fig 4A). The comparison between [Ca^2+^] 10 mM and [Ca^2+^] 0 mM contained numerous metabolic pathways that were influenced by the presence of calcium. Further comparison of the pathways up-regulated in [Ca^2+^] 10 mM relative to [Ca^2+^] 0.1 mM indicated that the most enriched pathways in [Ca^2+^] 10 mM were protein processing in endoplasmic reticulum, nitrogen metabolism, ABC transporters, and endocytosis (Fig 4A). In both cases, the down-regulated pathways were merely enriched compared with the up-regulated pathways in [Ca^2+^] 10 vs 0 mM (Fig 4B).

### Differentially expressed proteins at different calcium concentrations

**Fig 5:**
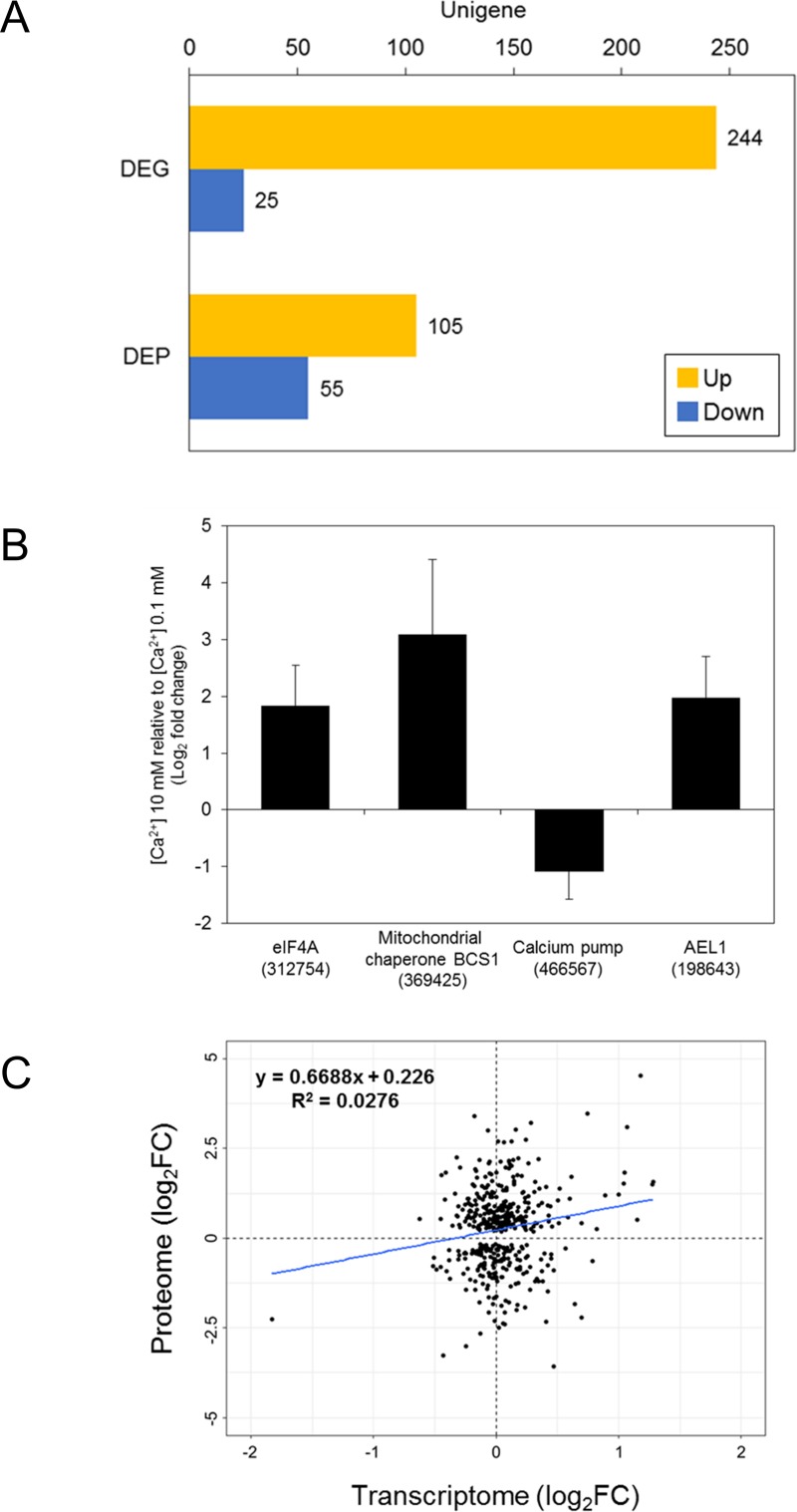
Unigenes differentially expressed in both the transcriptome and proteome. (A) The differentially expressed genes (DEGs) and proteins (DEPs) in [Ca^2+^] 10 mM relative to [Ca^2+^] 0.1 mM (yellow: up-regulated, blue: down-regulated). (B) The putative biomineralization-related DEPs (log_2_FC; ± SE): AEL1 (c31742_g1_i2), calcium pump (c31573_g1_i1), mitochondrial chaperone BCS1 (c23862_g2_i1), and eIF4A (c22513_g1_i1). (C) Correlation between differentially expressed transcripts and proteins.

To examine *E*. *huxleyi* cells cultivated at two different calcium concentrations ([Ca^2+^] 10 and 0.1 mM), we next analyzed the differentially expressed proteins (DEPs) (Table H in S1 File). As a reference, the identified peptide fragments were matched to the ORF sequences predicted from the RNA-seq data. The ORF sequences of 3,151 unigenes were matched with the sequenced peptides. Between [Ca^2+^] 10 mM and [Ca^2+^] 0.1 mM, the DEPs were filtered using | log_2_FC | > 1 with a P-value cut-off of 0.05. Among the DEPs, 105 ORF sequences were up-regulated, and 55 ORF sequences were down-regulated in [Ca^2+^] 10 mM relative to [Ca^2+^] 0.1 mM (Fig 5A). The GO level-4 categories of DEPs show that the GO terms ‘macromolecular metabolic process’, ‘anion binding’, and ‘cytoplasm’ were the largest terms from the biological process, molecular function, and cellular component categories, respectively (S7 Fig). In addition, a KEGG pathway enrichment analysis was performed for the DEPs up- and down-regulated in [Ca^2+^] 10 mM relative to [Ca^2+^] 0.1 mM (S8 Fig). The ‘aminoacyl-tRNA biosynthesis’, ‘ribosome biogenesis in eukaryotes’, ‘ribosome’, and ‘spliceosome’ pathways were up-regulated DEPs (S8A Fig). For DEPs down-regulated in [Ca^2+^] 10 mM relative to [Ca^2+^] 0.1 mM, the ‘spliceosome’ and ‘RNA transport’ pathways were enriched (S8B Fig). Furthermore, the putative biomineralization-related proteins were searched against the DEPs. The eIF4 (JGI ID 312754), putative mitochondrial chaperone BCS1 (JGI ID 369425), and AEL1 (JGI ID 198643) were up-regulated DEPs, and the putative calcium pump (JGI ID 466567) was a down-regulated DEP in [Ca^2+^] 10mM relative to [Ca^2+^] 0.1 mM (Fig 5B),

### Comparison of the KEGG pathways in the transcriptome and proteome analyses

**Fig 6:**
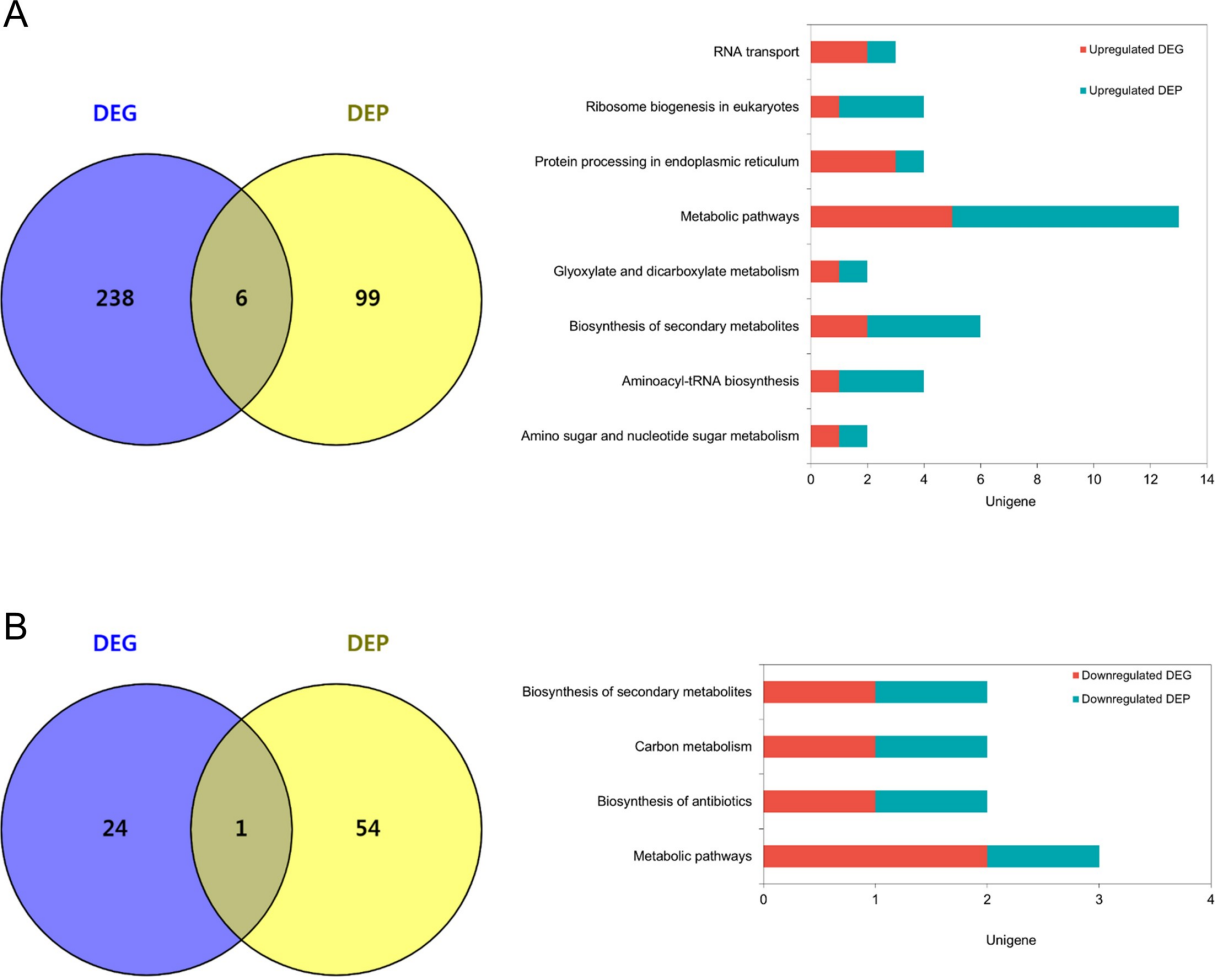
KEGG pathways in DEGs and DEPs of *E*. *huxleyi* at different calcium concentrations. (A) The up- and (B) down-regulated DEGs and DEPs involved in metabolic pathways of *E*. *huxleyi*. The numbers of common and unique DEGs and DEPs are shown in the Venn diagram. The bar chart shows the sum of the pathways commonly involved in DEGs and DEPs.

The transcriptome and proteome had a very low correlation coefficient (r = 0.1661) between the DEGs and DEPs (Fig 5C). Despite the low correlation, the pathways involved in both the DEGs and DEPs were identified using a KEGG pathway analysis. The pathways involved in the up-regulated DEGs and DEPs include metabolic pathways, biosynthesis of secondary metabolites, and protein processing in endoplasmic reticulum (Fig 6A, Table I in S1 File). The down-regulated groups were metabolic pathways, biosynthesis of secondary metabolites, carbon metabolism, and biosynthesis of antibiotics (Fig 6B, Table I in S1 File).

## Discussion

In this study, we report a *de novo* transcriptome analysis of calcifying *E*. *huxleyi* CCMP371 combined with a proteomic analysis profiling the molecular candidates related to calcium concentrations to identify putative biomineralization-associated genes. In addition to the biological importance of *E*. *huxleyi*, materials scientists are focusing on its potential as a biomineralized material for nanotechnology [40–42]. Therefore, our study advances current knowledge about the biomineralization of *E*. *huxleyi* which has an important ecological role and potential nanotechnology applications.

Herein, we have reported which gene expressions differ at different calcium concentrations in *E*. *huxleyi* CCMP371. Although coccolith formation was inhibited by low calcium concentrations, limiting the calcium concentration in the culture medium did not disturb the growth of *E*. *huxleyi* in the current study, which is consistent with previous reports [11–16]. In contrast, recent work by Walker et al. (2018) has shown that a low calcium concentration inhibited the growth of heavily calcified *Coccolithus braarudii* [43]. These two ecologically important species of coccolithophores, *C*. *braarudii* and *E*. *huxleyi*, thus exhibit distinct calcification requirements. Although *E*. *huxleyi* might not be representative of all coccolithophores, a draft genome of strain CCMP1516 has been sequenced, and foundational work to understand biomineralization at the molecular level has been done [5–11, 37]. Elucidating the complex molecular apparatus of coccolith formation will require a thorough understanding of the environmental conditions that modify the coccolith-forming ability. Thus, *E*. *huxleyi* is a suitable organism for our study of calcium concentration–related genes because its growth is unaffected by changes in calcium availability. Nonetheless, to understand the calcification process among coccolithophore species, especially those species for which molecular information is lacking, further genomic and transcriptomic analyses will be required.

### Functional analysis of DEGs in ambient- and limited-calcium conditions

The *de novo* assembly was performed to retrieve transcripts in the form of genome segments missing from the genome assembly [44]. The complex molecular network of calcification is regulated by various ion transporters, calcium-binding proteins, and membrane components. Our GO enrichment analysis of up-regulated DEGs at [Ca^2+^] 10 mM relative to [Ca^2+^] 0 mM indicates that integral membrane components are related to the crucial process associated with calcium concentration. The KOG enrichment analysis of up-regulated DEGs in [Ca^2+^] 10 mM relative to [Ca^2+^] 0 mM reveals two enriched functional clusters ‘secondary metabolites biosynthesis, transport, and catabolism’ and ‘signal transduction mechanism’. In the cluster of ‘secondary metabolites biosynthesis, transport, and catabolism’ unigenes associated with the ABC superfamily were notable. The ABC transporters form protein superfamily which is responsible for active transport of substrates across the membrane lipid bilayer [45]. Substrates such as ions, sugars, peptides, and other molecules that are mostly hydrophilic can be transported by ABC transporters. The intracellular transport of nutrients, especially ions, is crucial to the calcification of *E*. *huxleyi*. Calcium and bicarbonate ions are essential elements required inside the CV to initialize calcite formation. Consequently, the genes in the ‘secondary metabolites biosynthesis, transport, and catabolism’ cluster, which are enriched in the calcifying condition ([Ca^2+^] 10 mM) compared to the non-calcifying condition ([Ca^2+^] 0 mM), are likely related to the intracellular transport of nutrients.

The pathway involved in the phosphatidylinositol signaling system, which is important for signal transduction processes, including lipid signaling pathways in a calcium-dependent manner, was enriched in the GO analysis [46, 47]. Furthermore, among the up-regulated DEGs in [Ca^2+^] 10 mM relative to [Ca^2+^] 0 mM, the ‘signal transduction mechanism’ cluster (serine/threonine protein kinase, phosphoinositide-specific phospholipase C, and ion channels) was enriched in the KOG enrichment analysis. However, the specific signaling pathways involved in calcification remain unknown. Phosphatidylinositol signaling is a crucial signaling pathway that controls the intracellular calcium concentration by tightly regulating the cytosolic concentration of calcium ions [48]. Our analysis at different calcium concentrations indicates that phosphatidylinositol signaling is an intracellular signaling pathway associated with calcification.

The up-regulated DEGs in [Ca^2+^] 10 mM relative to [Ca^2+^] 0 mM indicated enrichment of various metabolic pathways, such as lipid metabolism and amino acid metabolism. Sphingolipid metabolism was identified as the most significantly enriched pathway in the KEGG analysis. The sphingolipids are known to regulate calcium signaling through various pathways [49]. However, our comparison between [Ca^2+^] 10 mM and [Ca^2+^] 0 mM reveals that the pathway is closely connected to the presence of calcium. Thus, the pathways enriched in [Ca^2+^] 10 mM relative to [Ca^2+^] 0 mM are probably closely related to calcium homeostasis and signaling.

To reduce the DEGs, including calcium signaling pathways, and unravel the genes putatively associated with calcification, we analyzed the KEGG pathways of DEGs between [Ca^2+^] 10 mM and [Ca^2+^] 0.1 mM. The pathways involved in ‘protein processing in endoplasmic reticulum’ and ‘ABC transporters’ were significantly enriched in [Ca^2+^] 10 mM relative to [Ca^2+^] 0.1 mM. The DEGs in the ‘protein processing in endoplasmic reticulum’, the molecular chaperone DnaK, a hypothetical protein (JGI ID 461210), eukaryotic translation initiation factor 2 subunit alpha (eIF2α) (JGI ID 366908), and ER-oxidoreductin-1 (Ero1) (JGI ID 461210) included in the pathways. The molecular chaperones help proteins reach their original shape, prevent protein aggregation, and refold misfolded proteins [50]. DnaK, the major bacterial chaperone hsp70 (70 kDa heat shock protein), is well conserved from prokaryotes to eukaryotes [51]. Two calcium ions can bind within the ATPase domain of hsp70 [52]. The eukaryotic translation initiation factors regulate the initiation phase of eukaryotic translation. During ER (endoplasmic reticulum) stress, eIF2α/activating transcription factor 4 (ATF4) signaling prevent a decrease in protein synthesis [53]. In chronic kidney disease (CKD), Masuda et al. (2013) reported that tumor necrosis factor-alpha induces the protein kinase RNA-like endoplasmic reticulum kinase-eIF2α-ATF4-C/EBP homologous protein signaling part of the ER stress response, causing CKD-dependent vascular calcification [54]. In eukaryotes, Ero1 catalyzes the formation and isomerization of protein disulfide bonds in the ER [55]. The translational attenuation caused by ER stress phosphorylates eIF2α to reduce the translation process. Also, under ER stress conditions, the expression of molecular chaperones is induced to increase the protein folding ability of the ER. In our study, *E*. *huxleyi* cultivated at ambient calcium concentration had relatively high expression of ER stress-related genes compared with the cells cultured at limited calcium concentration. Perhaps, coccolithophore calcification acts as an efficient mechanism to mitigate cellular calcium intoxication and cope with high external calcium concentrations [56]. Similarly, the putative genes identified from our previous study, eIF4A and putative ABC transporter, were up-regulated in the ambient calcium concentration relative to the limited calcium concentrations in the current transcriptome results (Fig 3) [16].

The genes included in the ABC transporter category were ATP-dependent bile acid permease (c30830_g1_i1, JGI ID 430601) and ABC transporter (c29057_g2_i1, JGI ID 430601). The ATP-dependent bile acid permease encoding gene ybt1 in yeast has been known to regulate the translocation of phosphatidylcholine to the vacuole lumen and the regulation of calcium homeostasis [57, 58]. Furthermore, as reported by Sviben et al. (2016), high concentrations of a disordered form of calcium stored in the vacuole-like compartment is directly associated with the CV [59]. Because the ion transport process is crucial for the formation of coccoliths in *E*. *huxleyi*, the unigenes enriched in this pathway are putative genes that directly or indirectly regulate the calcification process.

### Comparison between DEGs and DEPs

Our comparison results between the transcriptome and proteome for DEGs and DEPs showed a low correlation coefficient. Combining the transcriptome and proteome results is controversial because they generally show a low correlation [60–62]. Nonetheless, our results provide detailed information about the molecular mechanisms related to calcium at both the transcript and protein levels in *E*. *huxleyi*. Although the largest number of DEPs are annotated as hypothetical proteins and predicted proteins, DEPs related to calcification at different calcium concentrations need further investigation. We also performed a pathway analysis comparing DEGs and DEPs at different calcium concentrations. The up-regulated pathways involving the DEGs and DEPs were related to ‘protein processing in endoplasmic reticulum’ and the metabolic pathways indicated by the KEGG pathway analysis revealing higher ER-associated gene expression in the ambient calcium condition than in the limited calcium condition. Whether calcium is deposited in the vacuole-like reservoir or processed directly into the CV needs to be elucidated. Therefore, the complex intracellular process that emphasizes calcium, especially the genes or proteins related directly or indirectly to the calcification process, requires further study.

### Biomineralization-related genes at different calcium concentrations

In our transcriptome dataset, the putative genes thought to be involved in the biomineralization process were not consistent with the results of previous reports [10, 11, 37–39]. We examined 70 unique biomineralization-related genes (JGI IDs) with 98 unique homologous unigenes (Table G in S1 File). Of those 98 unigenes, 12.3% of unigenes were differentially expressed. Although the putative biomineralization genes were not significantly expressed in our dataset, the calcium-binding protein GPA was differentially expressed in [Ca^2+^] 10 mM relative to [Ca^2+^] 0 mM (Fig 2). The GPA protein is well known to be expressed in the CV [63]. That the expression of the GPA protein would vary with the calcium concentration is not surprising because of its calcium binding ability. However, in the results of Mackinder et al. (2011), GPA was ~10.8 fold down-regulated in [Ca^2+^] 10 mM compared with [Ca^2+^] 0 mM, which is opposite to our results. The *E*. *huxleyi* strain used by Mackinder et al. (2011) was calcifying CCMP1516, whereas we used CCMP371, but we cannot simply decide that the results are strain specific. The calcium homeostasis and calcification process is a complex system organized by the cell. Therefore, genetic manipulation is required to uncover the role of GPA in biomineralization. The two genes homologous to ‘fibrillins and related proteins contacting Ca^2+^-binding EGF-like domains (JGI ID 118025, 463266)’ were up-regulated in [Ca^2+^] 10 mM compared with [Ca^2+^] 0 mM. The ‘fibrillins and related proteins contacting Ca^2+^-binding EGF-like domains’ were also up-regulated under elevated *p*CO_2,_ which increased calcification [37]. In addition, our results indicated that the expression of these genes is up-regulated at the ambient calcium concentration (calcifying) compared with the limited calcium concentrations (non-calcifying). Therefore, we can carefully speculate that ‘fibrillins and related proteins contacting Ca^2+^-binding EGF-like domains’ are related to the calcification of *E*. *huxleyi* at different calcium concentrations.

Two inorganic carbon transporters (JGI ID 466232, 436956) were differentially expressed at the ambient calcium concentration compared with the limited calcium concentration, suggesting that these solute SLC4 transporters are related to the calcium concentration. In sea urchin embryos, the SLC4 family bicarbonate transporter regulates intracellular pH and biomineralization and is crucial for the production of an elaborate calcitic endoskeleton [64]. The increased SLC4 gene expression seen at the ambient-calcium concentration (10 mM) compared with the limited-calcium concentrations suggests its potential involvement in the calcification process. However, *E*. *huxleyi* showed increased calcification with long-term exposure to elevated temperature and *p*CO_2_, and the SLC4 transporters were not differentially expressed under those conditions [37]. Rokitta et al. (2012) reported that the SLC4 family bicarbonate transporter and plastid-targeted HCO_3_^-^ transporter were down-regulated under ocean acidification (OA) [65]. In another response to OA, the SLC family bicarbonate transporter was up-regulated in diploid *E*. *huxleyi* RCC1216. The bicarbonate transporters are thought to be localized in the plasma membrane and plastid. Therefore, it is difficult to relate all the HCO_3_^-^ transporters to the calcification process alone. However, understanding the calcium-dependent mechanism of calcification together with the OA response provides evidence that the SLC4 family of bicarbonate transporters is crucial to the mineralization process in *E*. *huxleyi*. The possibility that SLC4 transporters are involved in the calcification of *E*. *huxleyi* needs further assessment. Additionally, in cellular inorganic carbon fluxes, CAs play an important role in carbon acquisition. The putative delta CA (JGI ID 195575) has shown more than 5-fold up-regulated gene expression in the ambient calcium concentration compared with the limited calcium concentrations, suggesting a possible role in calcification.

Regulating intracellular pH is critical to calcification. In the giant clam, *Tridacna squamosa*, Na^+^/H^+^ exchangers play an important role in controlling increased H^+^ excretion during calcification [66]. From our results, the Na^+^/H^+^ exchanger (JGI ID 434034) was an up-regulated DEG at ambient calcium concentration relative to limited calcium concentration. While forming coccoliths inside the CV, production of H^+^ is necessary. The Ca^2+^/H^+^ antiporters and vacuolar H^+^-ATPase (V-ATPase) could be responsible for the removal of H^+^ produced during the calcification process [4]. Although the cellular pH homeostasis of *E*. *huxleyi* needs to be explored, our results suggest that the Na^+^/H^+^ exchanger is regulated by calcium concentration.

Furthermore, we have analyzed DEGs by mapping RNA-seq reads to the *E*. *huxleyi* reference genome (Table J in S1 File). The biomineralization-related DEGs, from the RNA-seq reads mapped to the reference genome (Table K in S1 File), showed correlation (R^2^ = 0.6527) with DEGs from the *de novo* transcriptome analysis. Biomineralization-related genes such as GPA (JGI ID 431830), bicarbonate transporter (JGI ID 436956), and Ca^2+^/Mg^2+^-permeable cation channel (JGI ID 460292) were noticeable. The DEPs from peptides mapped to the *E*. *huxleyi* reference sequences was analyzed as well (Table L in S1 File). Moreover, DEPs examined by mapping to the reference sequences showed correlation (R^2^ = 0.8914) with the DEPs analyzed based on the *de novo* transcriptome.

**Fig 7:**
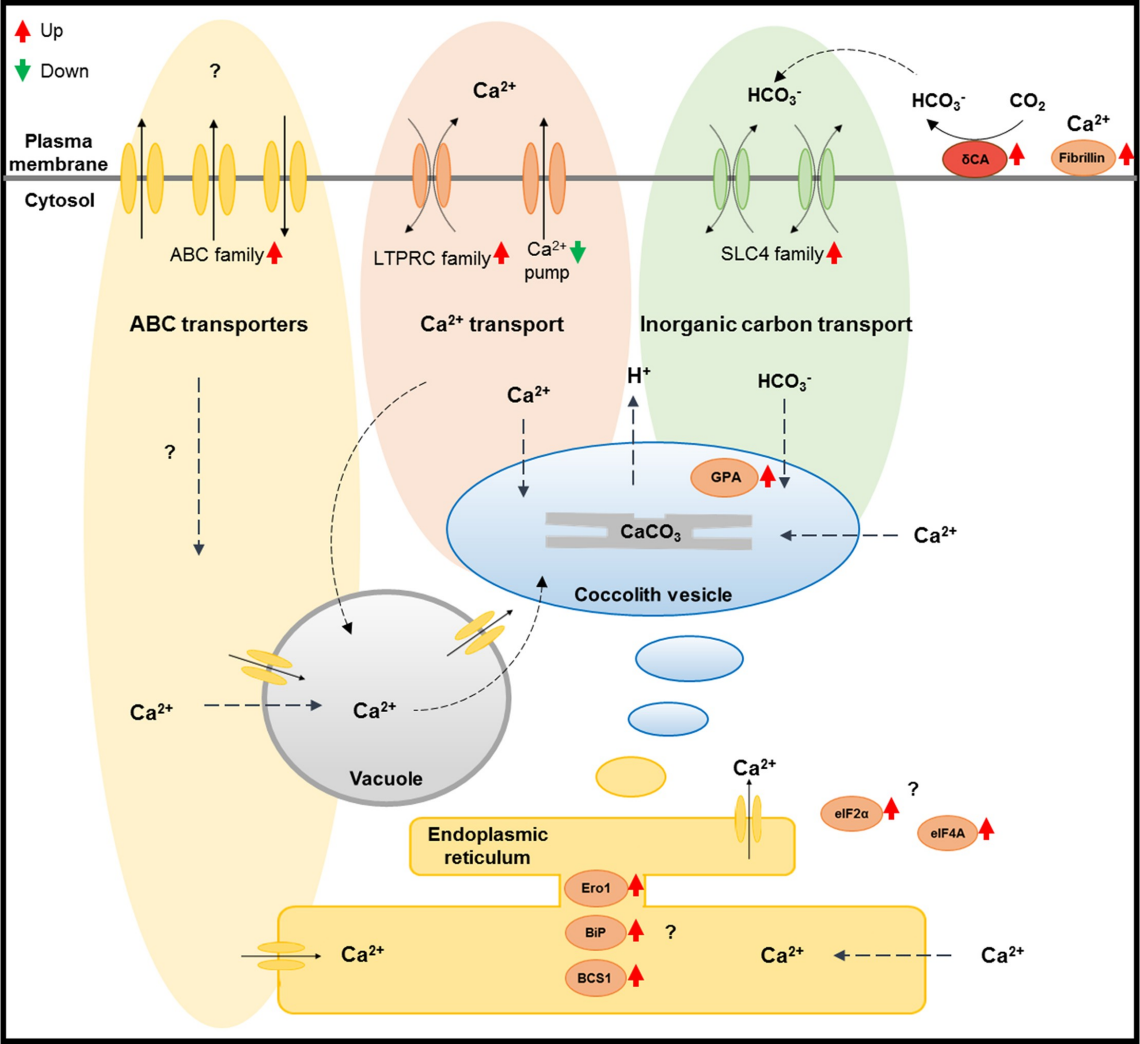
The hypothetical calcification-related intracellular model shown as a schematic cell diagram. This calcification-related model is suggested based on previously reported studies [4, 11, 59, 67]. The ABC family transporters (yellow, upper-left) were mostly included in the ‘secondary metabolites biosynthesis, transport, and catabolism’ of the KOG-enriched cluster. The LTRPC family cation channel (JGI ID 460292) and Ca^2+^-pump (JGI ID 466567) (red, upper-middle) were up- and down-regulated, respectively, at the ambient Ca^2+^ concentration relative to limited Ca^2+^ concentrations. Furthermore the HCO_3_^-^ transporter solute carrier 4 families (JGI ID 436956, 466232) (green, upper-right) are described in this study. The delta CA (JGI ID 195575) (dark red, upper-right) was the only up-regulated carbonic anhydrase in this study. The gene expression of the fibrillins and related proteins containing Ca^2+^-binding EGF-like domains (JGI ID 118025, 463266) (orange, upper right-hand corner) was also up-regulated at ambient Ca^2+^ concentration relative to the limited Ca^2+^ concentrations. The eukaryotic translation initiation factors (eIFs) (eIF4A: JGI ID 312754, eIF2α: JGI ID 366908) (orange, lower-right) were up-regulated at the ambient Ca^2+^ concentration relative to the limited Ca^2+^ concentrations as well. The eIFs have the potential to regulate the signaling pathway related to calcification at different calcium concentrations. The molecular chaperones (orange, lower-middle) BiP (JGI ID 442092), BCS1 (JGI ID 369425) and Ero1 (JGI ID 461210) are also possible factors in the calcification process.

In addition to the previous reports, we propose that these calcium transporters, inorganic transporters, and ABC family transporters are related to calcification at different calcium concentrations (Fig 7). The exact role of these transporters is uncertain, but their transportability and differential gene expression in our results suggest that they are involved in the calcification process. Consequently, we propose a hypothetical calcification-related intracellular model for *E*. *huxleyi* at different calcium concentrations (Fig 7).

In this study, we have reported more than 38,000 annotated transcripts that could supply valuable molecular information for future mineralization studies in *E*. *huxleyi*, especially related to calcium. Uncovering calcium-associated regulation at the molecular level increases understanding of the complex biomineralization process in the coccolithophorid alga *E*. *huxleyi*. In this context, establishing a transformation system for *E*. *huxleyi* is important to elucidate the regulatory genes involved in the biomineralization process. Unfortunately, no transformation techniques for *E*. *huxleyi* are available. However, such techniques have recently been successfully established for *P*. *carterae* [68] and *Tisochrysis lutea* [69]. Therefore, we hope to develop a variety of genetic tools and transformation systems for *E*. *huxleyi* to better understand the genetic function of this microorganism, including the biomineralization process.

## Supporting information

S1 Fig Transcriptome assembly assessment. (A) Benchmarking Using Single Copy Orthologues (BUSCO) and (B) overall read mapping ratio.
(TIF)

S2 Fig Functional annotation of assembled unigenes based on Gene Ontology (GO) classifications. (TIF)

S3 Fig Distribution of assembled unigenes using the euKaryotic Orthologous Groups (KOG) functional classification. The 10,692 unique sequences were divided into 26 KOG categories.
(TIF)

S4 Fig KEGG pathway classification of assembled unigenes. (TIF)

S5 Fig The differentially expressed genes at different calcium concentrations. Comparison between (left) [Ca^2+^] 0.1 vs 0 mM, (middle) [Ca^2+^] 10 vs 0.1 mM, and (right) [Ca^2+^] 10 vs 0.1 mM are shown by MA plot (| log_2_FC | > 1 are colored in blue).
(TIF)

S6 Fig The Gene Ontology (GO) terms in differentially expressed genes between different calcium concentrations. The level 4 GO terms of comparison between (A) [Ca^2+^] 10 vs 0 mM and (B) [Ca^2+^] 10 vs 0.1 mM are shown (Biological process: green, molecular function: blue, cellular component: yellow).
(TIF)

S7 Fig The Gene Ontology (GO) terms in differentially expressed proteins between different calcium concentrations. The level 4 GO terms of comparison between [Ca^2+^] 10 vs 0.1 mM are shown (Biological process: green, molecular function: blue, cellular component: yellow).
(TIF)

S8 Fig The KEGG pathway enrichment analysis in differentially expressed proteins between [Ca^2+^] 10 mM vs [Ca^2+^] 0.1 mM. The KEGG pathways enriched in (A) up- and (B) down-regulated DEPs.
(TIF)

S1 File Supplementary Tables. (A) Raw data statistics of RNA sequencing. (B) Primers used for qPCR. (C) Statistics of mapped reads to the *Emiliania huxleyi* reference genome (v1.0). (D) *De novo* transcriptome assembly statistics. (E) Overall annotations to public databases. (F) Differentially expressed genes at different calcium concentrations. (G) The putative biomineralization-related genes in DEGs and DEPs. (H) Differentially expressed proteins at different calcium concentrations. (I) The pathways involved in both DEGs and DEPs at different calcium concentrations. (J) Differentially expressed genes at different calcium concentrations analyzed by RNA-seq reads mapped to the *E*. *huxleyi* reference genome (v1.0). (K) The putative biomineralization-related DEGs analyzed by RNA-seq reads mapped to the *E*. *huxleyi* reference sequences (v. 1.0). (L) Differentially expressed proteins at different calcium concentrations analyzed by peptides mapped to the *E*. *huxleyi* reference sequences (v1.0).
(XLSX)
