# Supplementary figures and images for: De novo transcriptome profile of coccolithophorid alga Emiliania huxleyi CCMP371 at different calcium concentrations with proteome analysis
Source: PLoS One. 2019 Aug 29;14(8):e0221938. doi: 10.1371/journal.pone.0221938 (PMC6715215; doi:10.1371/journal.pone.0221938)

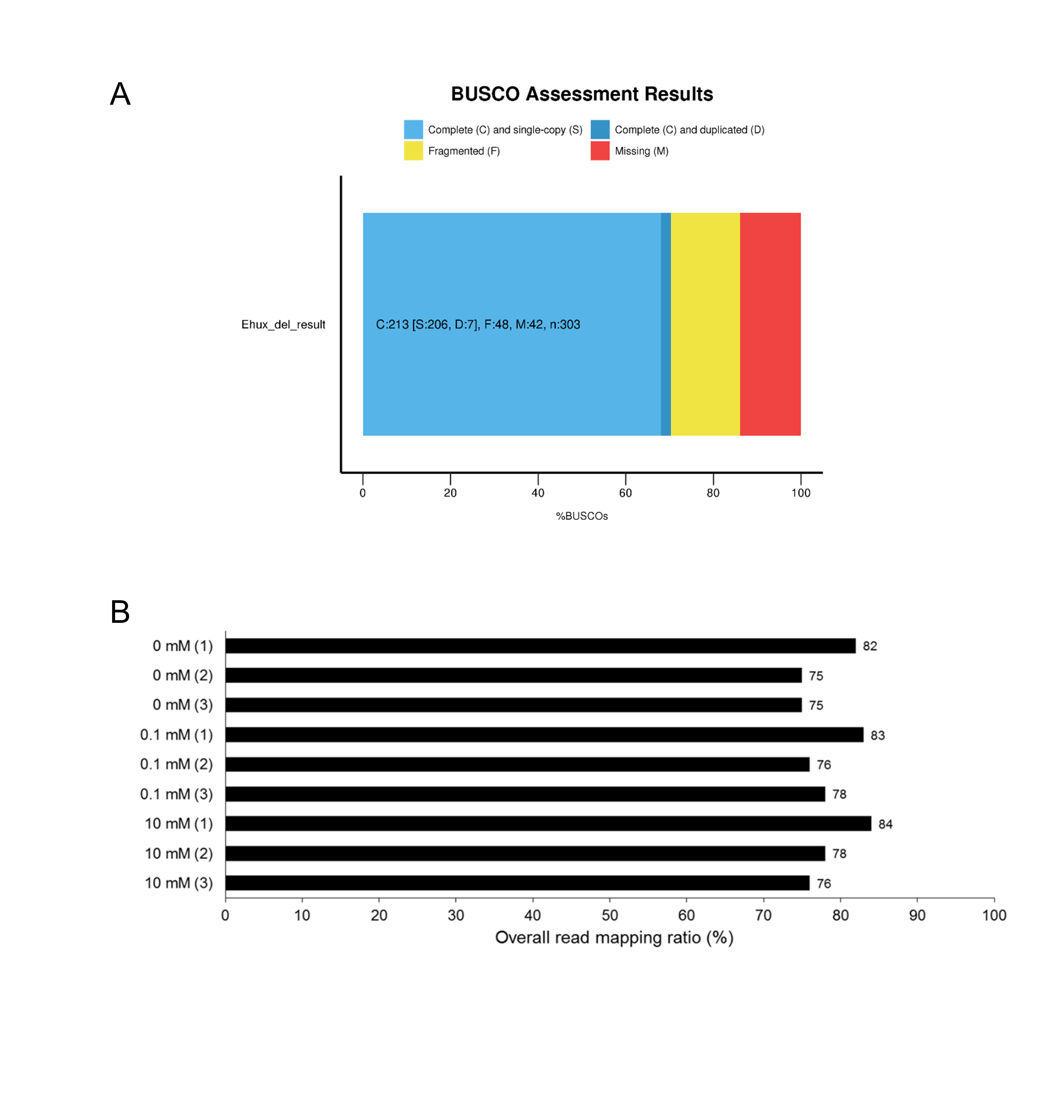

Supplement: S1 Fig — (A) Benchmarking Using Single Copy Orthologues (BUSCO) and (B) overall read mapping ratio. (TIF) [file pone.0221938.s001.tif]

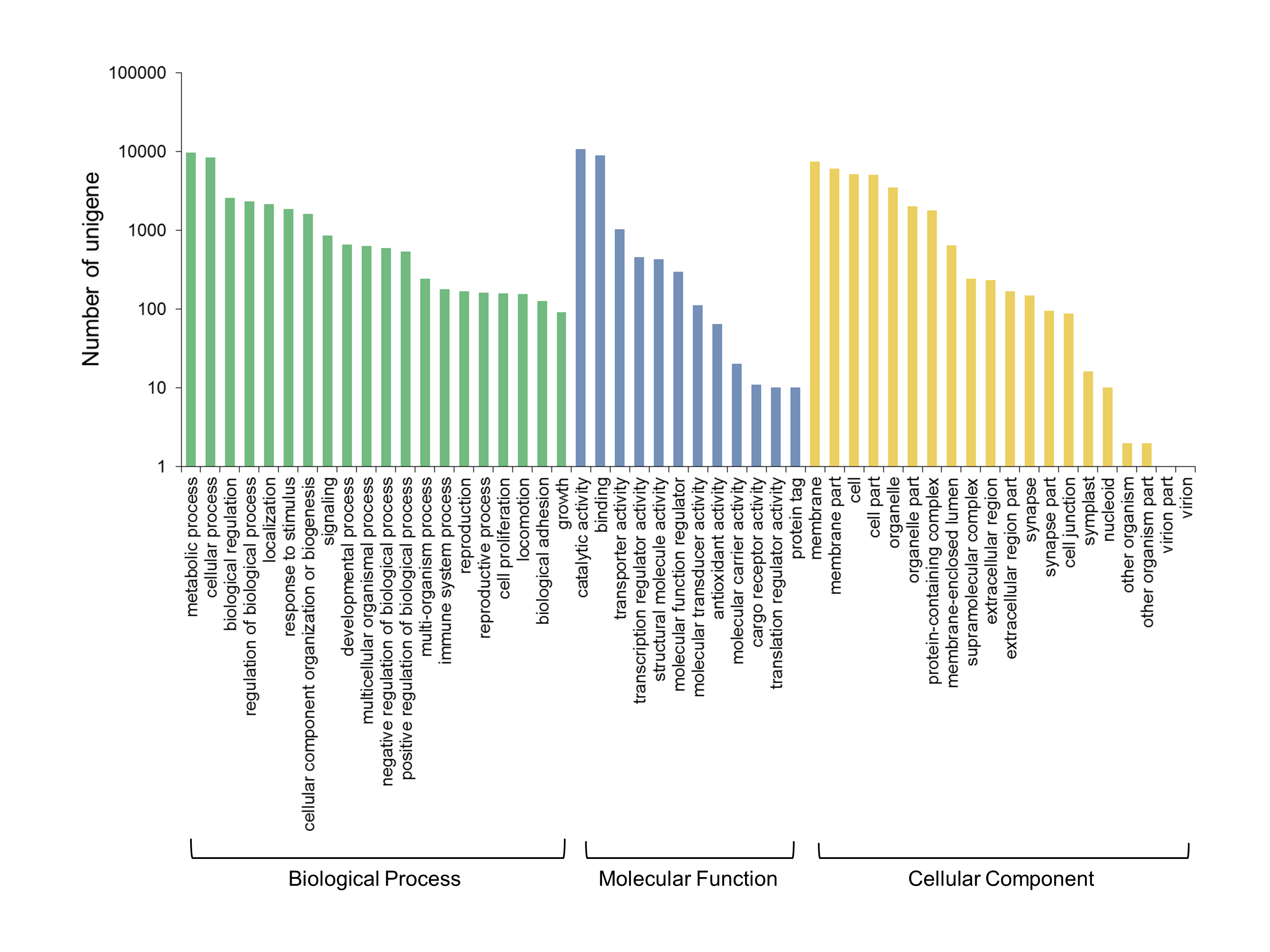

Supplement: S2 Fig — (TIF) [file pone.0221938.s002.tif]

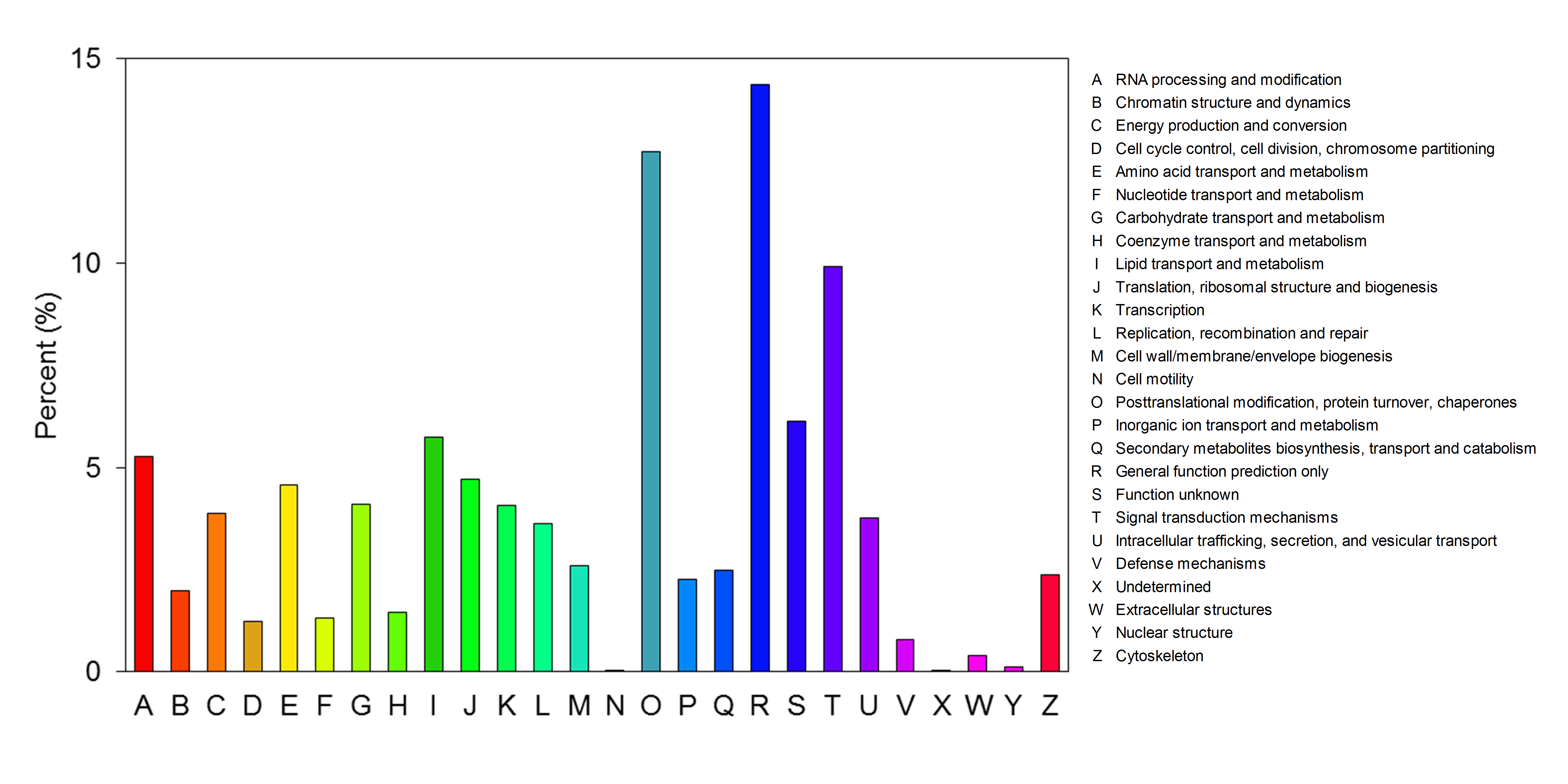

Supplement: S3 Fig — The 10,692 unique sequences were divided into 26 KOG categories. (TIF) [file pone.0221938.s003.tif]

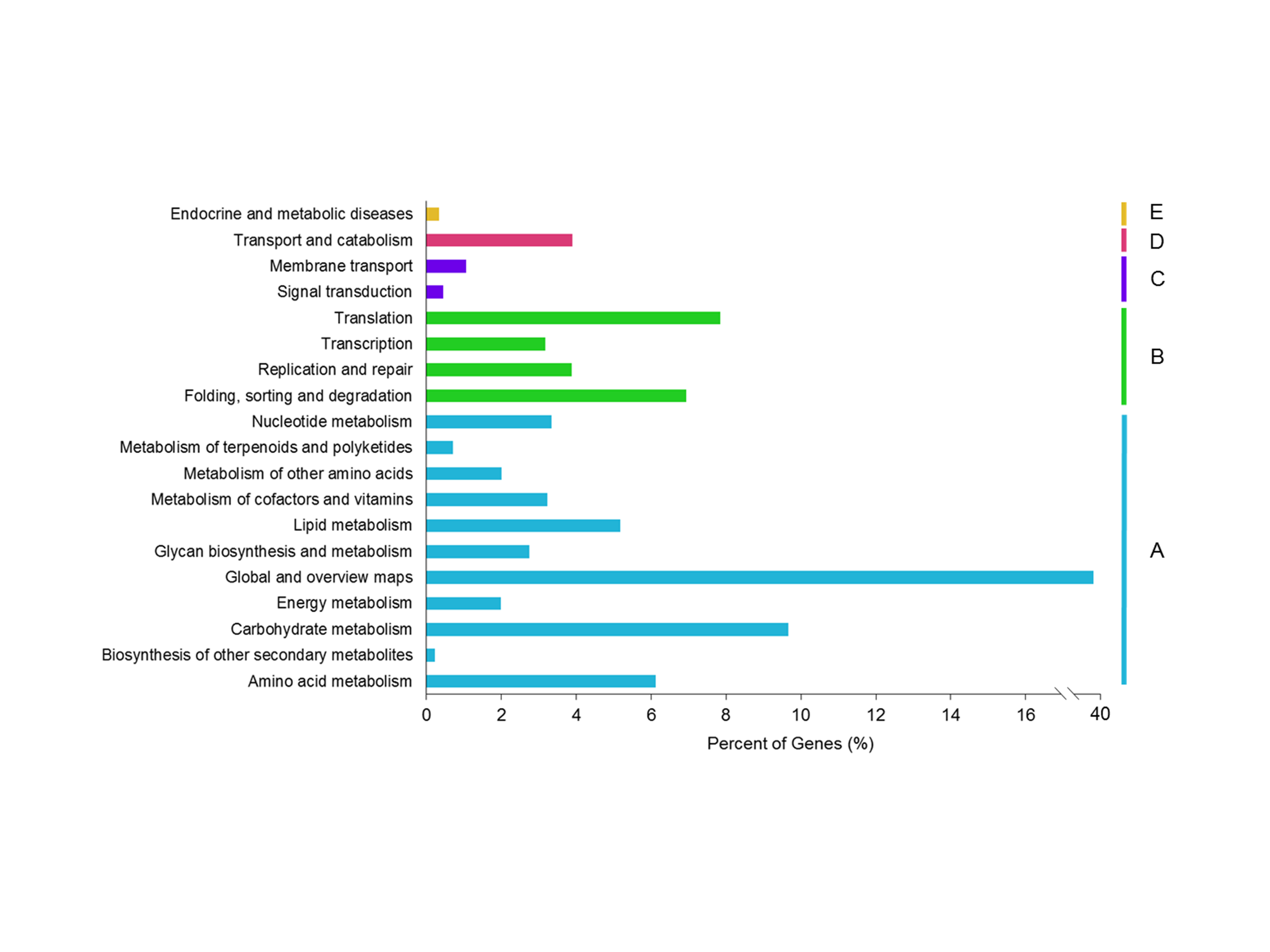

Supplement: S4 Fig — (TIF) [file pone.0221938.s004.tif]

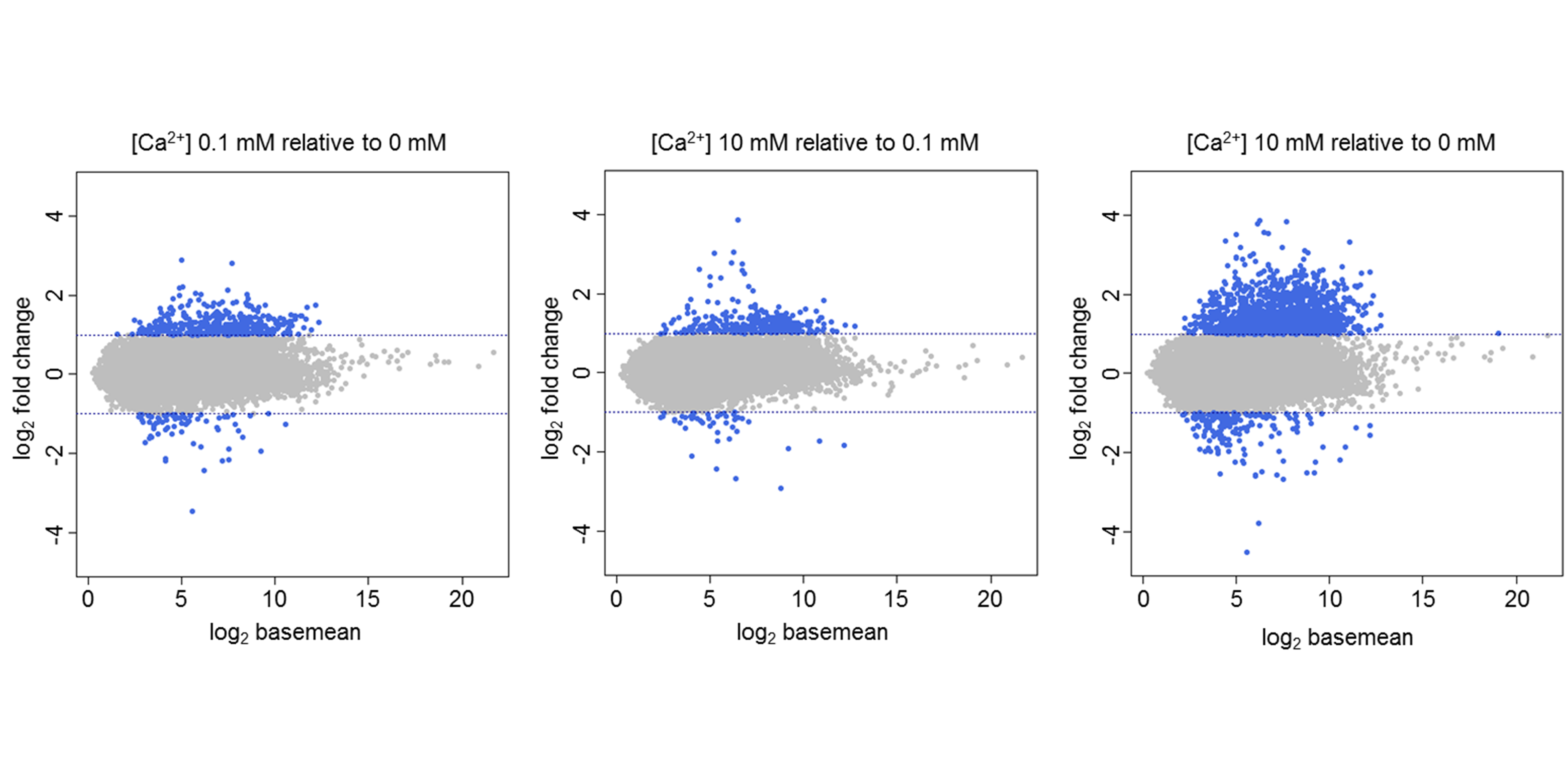

Supplement: S5 Fig — Comparison between (left) [Ca2+] 0.1 vs 0 mM, (middle) [Ca2+] 10 vs 0.1 mM, and (right) [Ca2+] 10 vs 0.1 mM are shown by MA plot (| log2FC | > 1 are colored in blue). (TIF) [file pone.0221938.s005.tif]

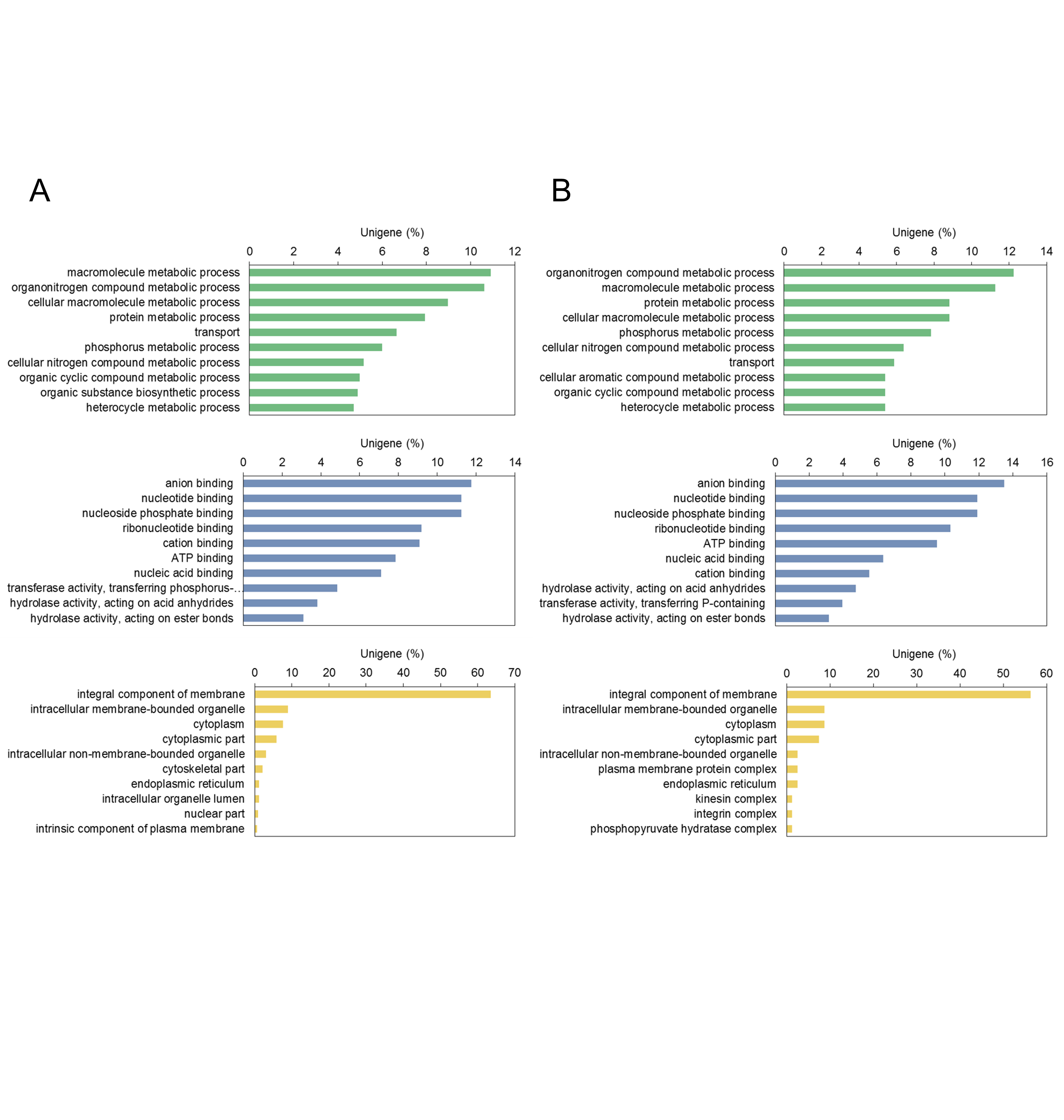

Supplement: S6 Fig — The level 4 GO terms of comparison between (A) [Ca2+] 10 vs 0 mM and (B) [Ca2+] 10 vs 0.1 mM are shown (Biological process: green, molecular function: blue, cellular component: yellow). (TIF) [file pone.0221938.s006.tif]

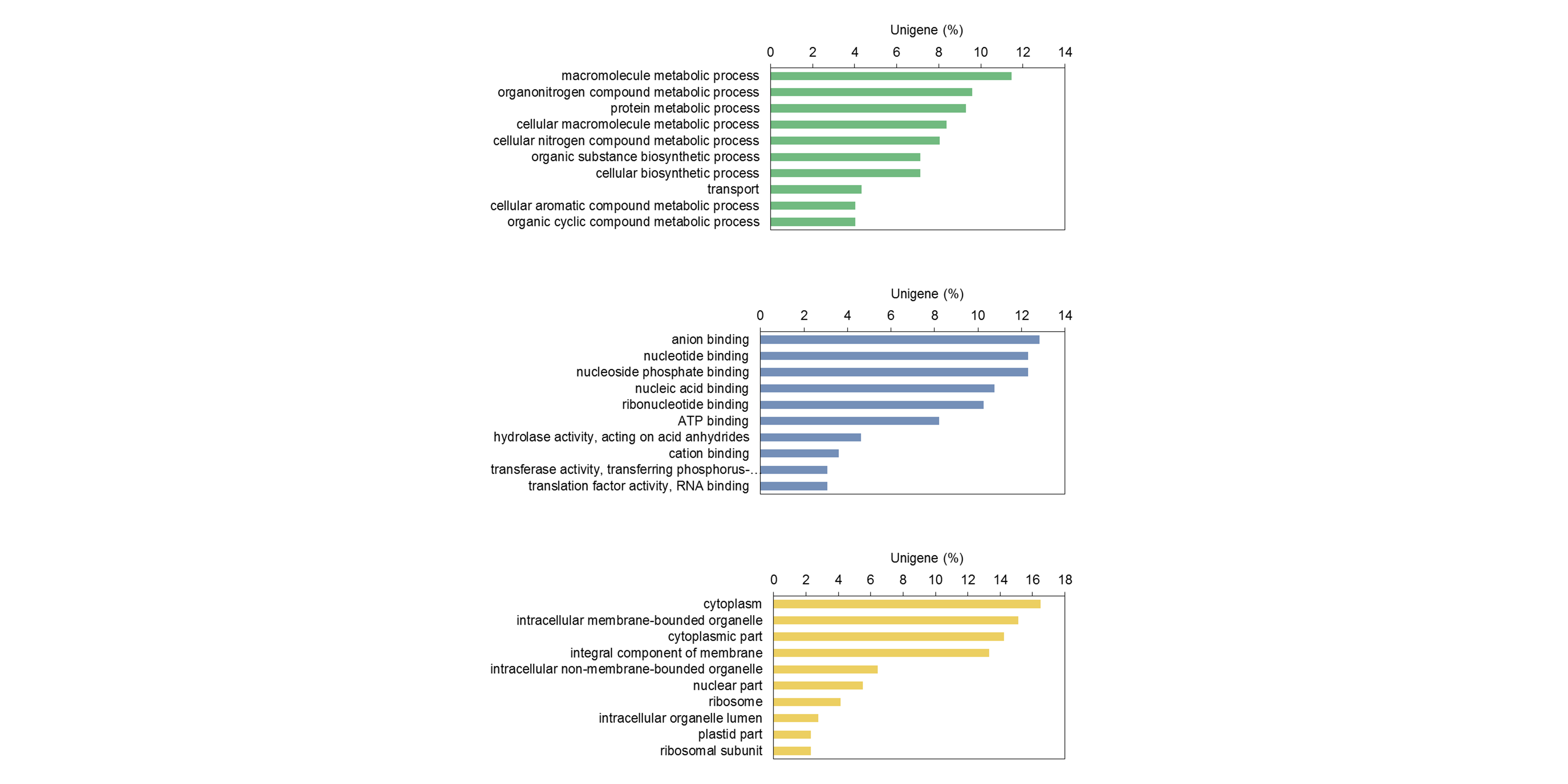

Supplement: S7 Fig — The level 4 GO terms of comparison between [Ca2+] 10 vs 0.1 mM are shown (Biological process: green, molecular function: blue, cellular component: yellow). (TIF) [file pone.0221938.s007.tif]

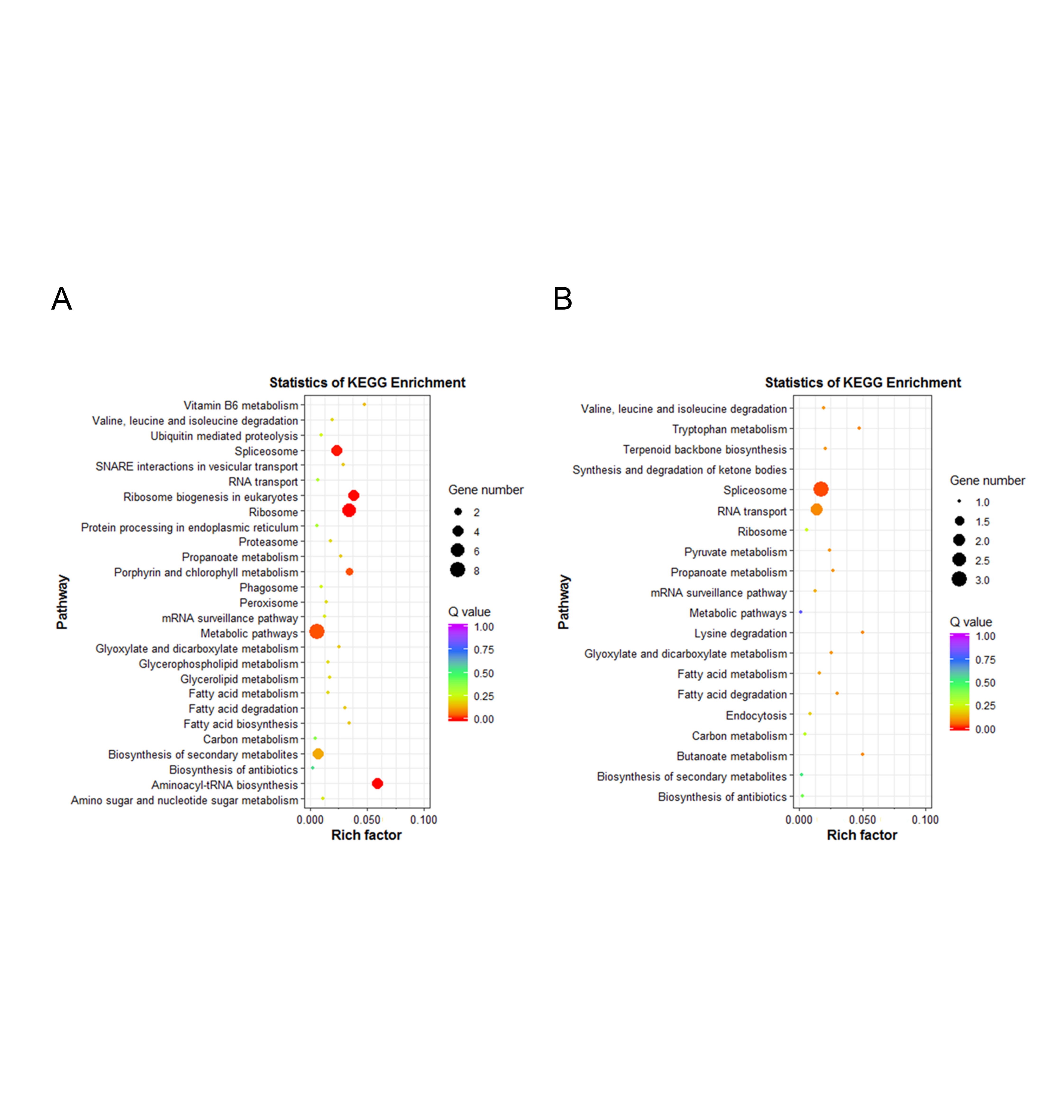

Supplement: S8 Fig — The KEGG pathways enriched in (A) up- and (B) down-regulated DEPs. (TIF) [file pone.0221938.s008.tif]
